# Towards repurposing the yeast peroxisome for compartmentalizing heterologous metabolic pathways

**DOI:** 10.1038/ncomms11152

**Published:** 2016-03-30

**Authors:** William C. DeLoache, Zachary N. Russ, John E. Dueber

**Affiliations:** 1 UC Berkeley and UCSF Graduate Program in Bioengineering, University of California, Berkeley (UC Berkeley), Berkeley, California 94720, USA; 2 Department of Bioengineering, UC Berkeley, Berkeley, California 94720, USA

## Abstract

Compartmentalization of enzymes into organelles is a promising strategy for limiting metabolic crosstalk and improving pathway efficiency, but improved tools and design rules are needed to make this strategy available to more engineered pathways. Here we focus on the *Saccharomyces cerevisiae* peroxisome and develop a sensitive high-throughput assay for peroxisomal cargo import. We identify an enhanced peroxisomal targeting signal type 1 (PTS1) for rapidly sequestering non-native cargo proteins. Additionally, we perform the first systematic *in vivo* measurements of nonspecific metabolite permeability across the peroxisomal membrane using a polymer exclusion assay. Finally, we apply these new insights to compartmentalize a two-enzyme pathway in the peroxisome and characterize the expression regimes where compartmentalization leads to improved product titre. This work builds a foundation for using the peroxisome as a synthetic organelle, highlighting both promise and future challenges on the way to realizing this goal.

## Abstract (web summary)

Compartmentalization of enzymes into cellular organelles is a promising strategy for improving pathway efficiency. Here, the authors use a high-throughput assay to identify enhanced peroxisomal targeting signals in yeast, and study the effects of peroxisomal compartmentalization on the performance of a model pathway.

## Graphical abstract

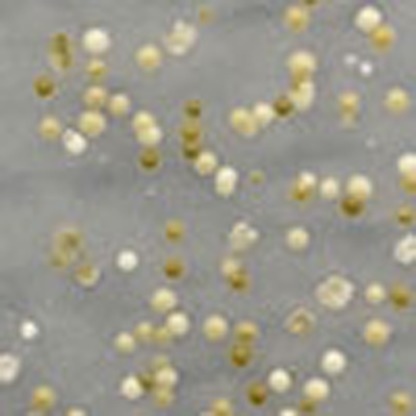

Metabolic engineering of microorganisms promises to enable the environmental friendly production of fuels, bulk chemicals and therapeutics^1^. A chief consideration for optimizing production in microbial hosts is a limiting crosstalk between high-flux engineered metabolic pathways and the native cellular processes of the production host^2^. Eukaryotes address the problem of metabolic crosstalk by partitioning proteins and metabolites in membrane-bound organelles to sequester toxic compounds^3^, direct the activity of enzymes towards specific substrates^4^, and establish distinct chemical environments (for example, altered pH or redox state^5^,^6^). Recently, metabolic engineers have begun to harness the subcellular organelles of *Saccharomyces cerevisiae* to take advantage of these properties: the mitochondrion was used to enhance the production of isobutanol^7^, and the vacuole was used as a site for methyl halide synthesis^8^. These successes relied on substrates that naturally accumulate in the mitochondria and vacuole, limiting the applicability to new pathways. We sought to extend the benefits of compartmentalization to any pathway of interest by laying the foundation for a flexible synthetic organelle.

A generalizable organelle compartmentalization strategy would feature several important traits: (i) the organelle should be orthogonal to native cellular processes, (ii) import of heterologous enzymes should be rapid, efficient and modular and (iii) transport of metabolites across the organelle membrane should be characterized and ultimately controllable. With the first criterion as a prerequisite for further engineering, we identified the yeast peroxisome as a promising starting point for the construction of such a synthetic organelle. The peroxisome of *S. cerevisiae* is primarily involved in β-oxidation of long-chain fatty acids, and previous characterization has shown that, unlike other organelles, peroxisome biogenesis can be completely disrupted without adversely affecting cell growth in common glucose-fed fermentation conditions^9^. Thus, the peroxisome can be cleared of its endogenous matrix proteins to establish an orthogonal subcellular compartment. We therefore focused on exploring the design rules for both protein and metabolite transport across the peroxisomal membrane as a step towards repurposing this organelle for heterologous pathway compartmentalization.

The wide diversity in peroxisomal form and function observed in nature provides further evidence for peroxisomes’ versatility and suitability as a synthetic organelle. Methylotrophic yeasts such as *Pichia pastoris* and *Ogataea polymorpha* possess peroxisomes that can expand up to 80% of the total volume of the cell^10^, suggesting that enormous protein capacity is achievable. Furthermore, the peroxisome has found use in nature not only as a catabolic compartment, but also as a site for unusual biochemistry, such as penicillin biosynthesis in *Penicillium chrysogenum*^11^ and light generation in the firefly *Photinus pyralis*^12^. Even diversity in peroxisomal structure has been observed, as import of HEX proteins transforms peroxisomes into Woronin bodies, which help plug leaks in damaged ascomycetes^13^. Peroxisomes have already attracted some interest from metabolic engineers, who have placed lycopene and polyhydroxyalkanoate biosynthetic pathways in the peroxisomal lumen^14^,^15^. However, these efforts left many open challenges, including validation and optimization of protein import, measurements of peroxisomal permeability and verification that intermediates are trapped within the peroxisome. It is these open challenges that we hope to address in this work.

Although the optimal parameters for peroxisomal protein import remain an open question, the biological mechanism has been extensively studied^16^,^17^,^18^. Peroxisomal matrix proteins generally contain one of two peroxisomal targeting signals (PTS1 or PTS2) that are recognized in the cytosol by corresponding receptor proteins, Pex5p and the Pex7p/Pex18p/Pex21p complex, respectively^16^. Upon recognition, these proteins are recruited to the Pex13p/Pex14p/Pex17p import pore complex and translocated into the peroxisomal lumen while remaining in the folded state^16^. The majority of native cargo proteins enter via some variant of the PTS1 tag, which canonically consists of Ser-Lys-Leu (SKL) at the carboxy-terminus^19^. Numerous studies have demonstrated that fusion of this three-amino-acid tag is sufficient to redirect cytosolic proteins into the peroxisome, however, recent work suggests that additional upstream residues also contribute to recognition by Pex5p^19^,^20^,^21^. A systematic experimental analysis of their effect on import has yet to be performed.

Although the participants involved in peroxisomal protein transport are well established, transport of metabolites into and out of the peroxisome remains a matter for debate. To date, few peroxisomal membrane transporters have been identified in yeast, namely Pxa1p/Pxa2p for fatty acids^22^ and Ant1p for ATP/AMP^23^. These transporters cannot account for the many metabolites consumed and released by the peroxisome. Early *in vitro* work suggested broad peroxisomal permeability to metabolites^24^, potentially accounting for the dearth of identified transporters. This view was challenged by *in vivo* studies that suggested fatty acyl-Coenzyme A molecules do not freely cross the peroxisomal membrane^25^. To reconcile these observations, a nonspecific, aqueous pore was proposed that allows for the free diffusion of molecules below a certain size^26^. Although recent *in vitro* results support this conclusion^23^, the picture is complicated by the possibility that the *in vitro* assay protocols themselves generate different pore sizes depending on peroxisome pretreatment^16^,^27^,^28^. *In vivo* validation is lacking, and the hypothesized protein responsible for this porosity remains unidentified in yeast.

Here, we extend the current understanding of peroxisome biology to uncover key design rules for compartmentalizing heterologous pathways in the yeast peroxisome. We develop a novel enzyme-based strategy for evaluating the efficiency of targeting non-native cargo to the peroxisome. Our assay, which we find to be more sensitive than fluorescence microscopy for assaying peroxisomal import, indicates that the canonical PTS1 tag is context-dependent and can result in slow import of cargo if the residues immediately upstream of the tag are not optimized. By screening a library of randomized PTS1 peptide linker sequences, we identify linker charge as an important factor and define a modular PTS1 tag with enhanced import performance. This enhanced tag allows efficient compartmentalization of several non-native enzymes and as well as the first *in vivo* demonstration that the peroxisomal membrane is indeed permeable to small metabolites while presenting a barrier to larger solutes. We take advantage of this size-dependent permeability to compartmentalize in the peroxisome a two-enzyme model pathway that consists of a small, permeant substrate and a larger, impermeant intermediate. Finally, we assess the effects of peroxisomal compartmentalization on pathway performance and identify future avenues for developing the peroxisome as a generalizable metabolic engineering tool.

## Results

### Development of a sensitive peroxisomal protein import assay

**Figure 1:**
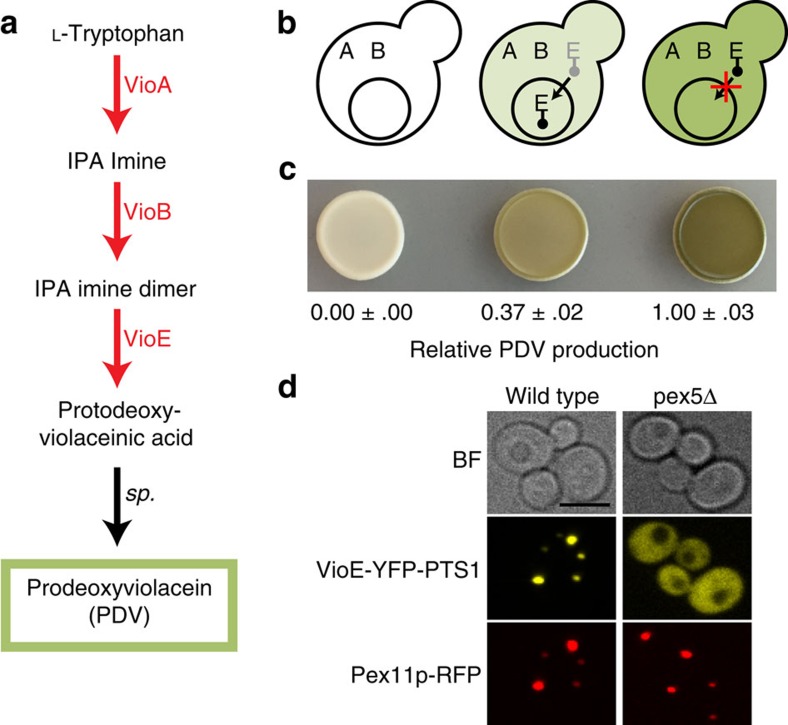
An enzyme-linked assay for peroxisomal import efficiency reveals cytosolic activity of PTS1-targeted cargo. (**a**) The green pigment PDV is produced from L-tryptophan as the result of three enzymatic steps (red arrows) and one spontaneous reaction (black arrow). (**b**) The precursors of PDV are colourless, so VioA and VioB can be freely expressed in the cytosol of yeast without any colour change (left). Efficient PTS1 targeting of VioE will result in enzyme sequestration in the peroxisome and considerably reduced PDV production in a wild-type strain (middle) compared with a *pex5*Δ strain that is deficient for PTS1 import (right). (**c**) Agar plate spots corresponding to the strains depicted above after 36 h of growth. VioE included a C-terminal YFP fusion and the canonical PTS1 tag (SKL). HPLC quantification of relative PDV production is included below each spot. (**d**) Fluorescence microscopy of yeast cells coexpressing the peroxisomal marker Pex11p-RFP and the VioE-YFP-PTS1 fusion protein in either a wild-type or *pex5*Δ strain background. Scale bar, 10 μm.

The first step in expressing a heterologous pathway in the peroxisome is ensuring that each enzyme is effectively compartmentalized. Although protein import is commonly monitored using fluorescence microscopy, we sought a method that would directly test the primary metric of interest for compartmentalizing new enzymatic pathways—cytosolic activity of an enzyme before peroxisomal import. For this purpose, we turned to a three-enzyme pathway from the bacterium *Chromobacterium violaceum* that converts tryptophan to the green pigment prodeoxyviolacein (PDV^29^; Fig. 1a and Supplementary Fig. 1). We reasoned that by expressing the first two pathway enzymes (VioA and VioB) in the cytosol but targeting the final enzyme (VioE) to the peroxisome, we would isolate VioE from its substrate, indole-3-pyruvic acid (IPA) imine dimer, and measurably reduce pathway flux. A large difference in PDV production between strains with VioE in the cytosol versus localized to the peroxisome would be indicative of efficient protein import (Fig. 1b).

To achieve peroxisomal targeting, we fused the canonical PTS1 tag (SKL) to the C-terminus of VioE, preceded by a yellow fluorescent protein (YFP) so that proper localization could be confirmed by fluorescence microscopy. We expressed all three enzymes in either a wild-type strain or a *pex5*Δ strain that is deficient for PTS1 protein import (Fig. 1c). Proper targeting of VioE to the peroxisome was confirmed by co-localization of VioE-YFP-PTS1 with a red fluorescent protein (RFP) peroxisomal marker, Pex11p-RFP (Fig. 1d). Although there was no cytosolic fluorescence observed via microscopy when VioE-YFP-PTS1 was expressed in wild-type cells, we measured only a 63% reduction in PDV production relative to the *pex5*Δ cytosolic control strain. This result suggested that, despite observing clearance of protein fluorescence from the cytosol, VioE remained in the cytosol long enough to confer significant activity before peroxisomal import. Reducing the occurrence of pre-import cytosolic activity is critical for ensuring that pathway activity is completely confined to the peroxisome.

### Positively charged residues enhance PTS1 import

Based on previous work showing that the residues adjacent to the PTS1 tag can affect protein import^20^, we attempted to improve compartmentalization of VioE by modifying a peptide linker sequence between the enzyme and the PTS1 tag. We took advantage of the high-throughput nature of our import assay to screen a library of randomized linker sequences inserted immediately preceding the carboxy-terminal SKL residues. This library was made up of six DNK degenerate codon repeats designed to balance the frequency of positively and negatively charged residues and to reduce the frequency of stop codons (Supplementary Fig. 2). When this VioE-YFP-6X_DNK-SKL library was transformed into a strain expressing VioA and VioB in the cytosol, we observed colonies that ranged in colour from white to dark green—presumably the result of differences in VioE peroxisomal import efficiency (Supplementary Fig. 3).

**Figure 2:**
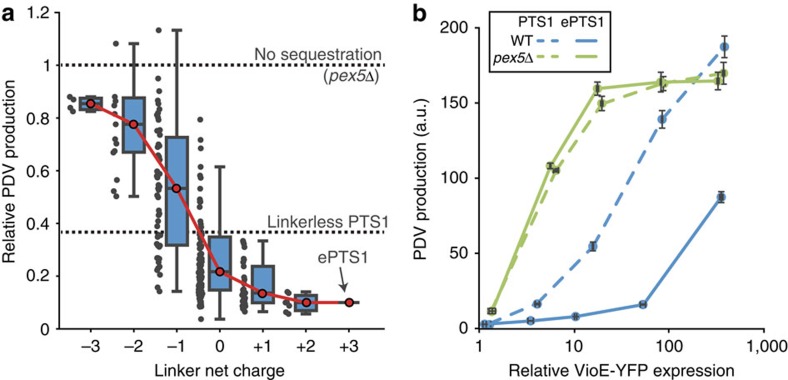
Library screening indicates that positively charged linker residues enhance PTS1-based import of VioE. (**a**) A randomized library of six amino-acid linkers was inserted between VioE-YFP and the PTS1 tag and coexpressed with cytosolically localized VioA and VioB. Individual data points represent PDV-linked fluorescence measurements of two hundred arbitrarily selected library members (excluding two outliers with values 1.45 and 1.73 at the −2 charge level for clarity), normalized relative to a *pex5*Δ strain and grouped by net charge of the linker residues. Quartiles and extrema for each net charge level are shown with a box-and-whiskers plot. The median PDV productivity at each net charge is further highlighted with a red line. Efficient peroxisomal import of VioE is correlated with decreased PDV production. We selected the sole +3 charge sequence, LGRGRR-SKL, for further study as ePTS1. (**b**) HPLC quantification of PDV from yeast strains expressing increasing levels of VioE-YFP fused to either naïve PTS1 (dashed lines) or ePTS1 (solid lines). Both wild-type (blue lines) and *pex5*Δ (green lines) strain backgrounds coexpressed VioA and VioB cytosolically. Error bars represent the mean±s.d. of four biological replicates.

To gain insight into how linker sequences affect VioE targeting, we sequenced the linker region of randomly picked colonies from the library transformation plate, obtaining sequences for 200 unique clones after removal of those with stop codons in the linker. To quantify PDV production in these strains, we developed a medium-throughput, microplate-based method for assaying PDV pathway flux. This method takes advantage of our observation that cells producing PDV are highly red fluorescent, presumably due to fluorescence of either PDV or a PDV derivative. After confirming that fluorescence measurements of extracts yielded an excellent linear correlation with HPLC-measured PDV production across a wide range of concentrations (Supplementary Fig. 4), we used this method to quickly screen our 200-linker library members (Supplementary Table 1). Analysis of these data showed a correlation between a linker’s net charge and the amount of PDV produced (*R*^2^=0.54 and standard error of regression (SER)=0.1928 when fit to a logistic curve; Fig. 2a). In general, clones with basic residues (Arg or Lys) in their linker produced low levels of PDV, indicating efficient targeting of VioE to the peroxisome. The correlation improved when we doubled the weight applied to the charge of the three linker residues immediately preceding the canonical PTS1 tag (*R*^2^=0.61 and SER=0.1781), establishing charge of the residues closest to SKL as a major determinant of import efficiency.

### A modular-enhanced PTS1 tag enables rapid peroxisome targeting

In search of a generalizable strategy for achieving efficient enzyme targeting, we decided to further characterize a single linker, LGRGRR, which appeared in one of our lowest PDV-producers and contains three basic residues (Fig. 2a). We named the combined linker-PTS1 tag (LGRGRR-SKL) ePTS1 for ‘enhanced PTS1’. To compare how much VioE could be sequestered with ePTS1 or the canonical PTS1 tag before high levels of cytosolic activity could be detected, we again fused these tags to VioE-YFP and performed enzyme titrations using a set of five promoters that span approximately three orders of magnitude in expression strength^30^ (Fig. 2b and Supplementary Fig. 5). These promoters are, from weakest to strongest, pREV1, pRNR2, pRPL18B, pTEF1 and pTDH3, with the last four promoters representing ∼4X, 12X, 45X and 140X the expression strength of pREV1. At the moderate pRPL18B expression level used earlier (Fig. 1), ePTS1 yielded a 95% reduction in PDV compared with a 63% reduction with PTS1 (Fig. 2b) relative to their corresponding *pex5*Δ control strains. When VioE was expressed under the strongest yeast promoter (pTDH3), the ePTS1 tag still reduced PDV production by half, whereas the PTS1 tag was saturated to the point of being indistinguishable from its *pex5*Δ control. In total, these results indicated that the ePTS1 tag directed VioE to the peroxisome far more efficiently than did the canonical PTS1 tag.

**Figure 3:**
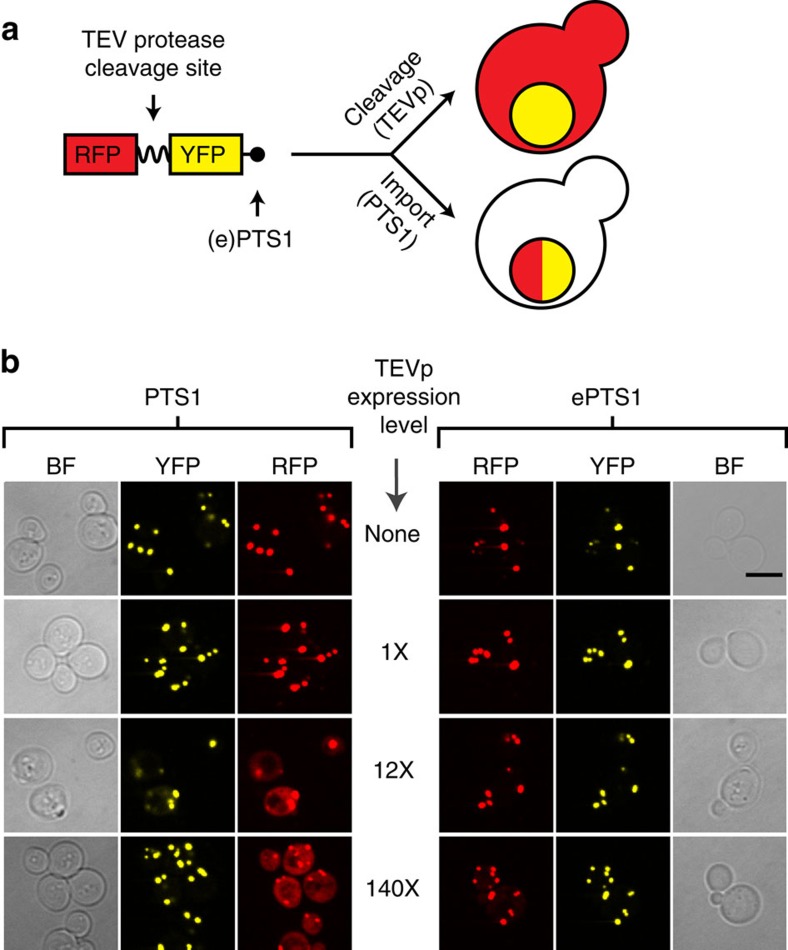
ePTS1-mediated peroxisomal import outpaces cytosolic proteolysis. (**a**) An assay for the relative speed of peroxisomal import was devised in which TEV protease-mediated protein cleavage competes with PTS1-mediated peroxisomal protein import. The RFP–YFP fusion protein shown here experiences two possible fates: either the fusion protein is cleaved before import, resulting in a diffuse red fluorescence, or peroxisomal import occurs before cleavage, resulting in punctate red fluorescence. In either case, yellow fluorescence is punctate, as peroxisomal import of YFP is not affected by proteolysis. (**b**) Brightfield (BF) and confocal fluorescence microscopy of yeast cells constitutively expressing an RFP-YFP-PTS1 or -ePTS1 fusion along with varying levels of TEV protease fused to CFP for quantification. The relative expression of TEV protease includes no TEVp, a baseline level (1X), as well as 12-fold and 140-fold above that baseline. Scale bar, 10 μm.

Although this VioE titration validated ePTS1’s utility for efficiently compartmentalizing large quantities of heterologous enzyme in the peroxisome, we wondered whether the rate of VioE import was responsible for the improved performance of the ePTS1 tag. To test the rate of cargo import, we designed a competition experiment that pitted peroxisomal import of a cargo protein against proteolysis of that same cargo by a cytosolically expressed protease (Fig. 3a). Our cargo was an RFP–YFP fusion protein with a tobacco etch virus (TEV) cleavage site inserted between the two fluorescent proteins and a PTS1 or ePTS1 tag fused to the C-terminus. By cytosolically expressing TEV protease (TEVp), we could gauge the speed with which the cargo was imported, as slow import would allow TEV site cleavage and cytosolic accumulation of RFP. Titrating TEV protease expression permitted us to modulate the rate of TEV site cleavage.

Our results showed the ePTS1-tagged cargo was imported much faster than cargo targeted via the canonical PTS1 (Fig. 3b). At the highest expression level of TEV protease, the mean RFP brightness in the cytosol (approximating protein concentration) was one-fourth of the peroxisomal brightness with the PTS1 tag, whereas ePTS1 showed a mean cytosolic brightness that was ∼50-fold lower than peroxisomal brightness. Based on the volumes of the two compartments^31^, ∼3% of the total RFP was imported before cleavage when using PTS1, versus 30% with ePTS1. Western blot analysis detected no difference between PTS1 and ePTS1 with respect to the fraction of cleaved cargo at any TEVp expression level (Supplementary Fig. 6), but the assay was likely confounded by TEV protease ‘piggybacking’ into the peroxisome via transient association with its substrate^32^,^33^ and cleaving the RFP–YFP inside the peroxisome.

We suspected that greater affinity for the import receptor Pex5p played a role in the increased speed of import with ePTS1 versus PTS1. By subjecting a C-terminal truncation of Pex5p and synthetic PTS1 peptides to *in vitro* binding analysis via fluorescence anisotropy, we found a 200-fold difference in affinity between the ePTS1 peptide (LGRGRRSKL) and a peptide of equal length that results from fusion of the canonical PTS1 to the C-terminus of YFP (DELYKGSKL) (Supplementary Fig. 7). The dissociation constant for the ePTS1 peptide was 0.8±0.1 μM, compared with 175.6±17.3 μM for the PTS1 peptide. These affinities were weaker than expected based on previous measurements^19^, but this discrepancy could be attributed to our use of fluorescein-tagged peptides instead of lissamine-tagged or our use of *S. cerevisiae* Pex5p instead of human Pex5p. Regardless, the large difference in PTS1 and ePTS1 affinity for Pex5p provides some evidence for the mechanism behind the improved efficiency of ePTS1.

**Figure 4:**
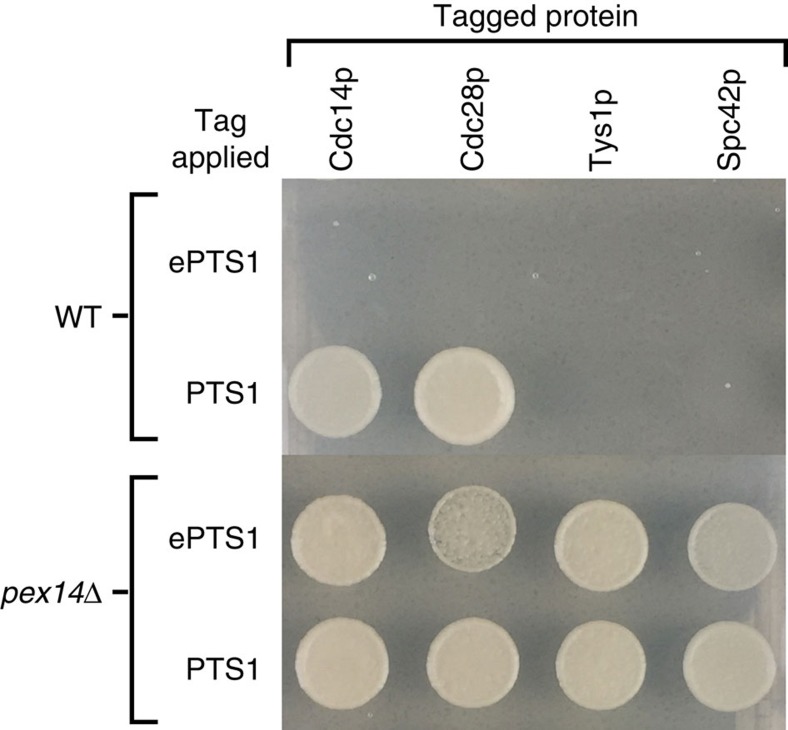
The ePTS1 tag modularity allows for rapid sequestration of a variety of proteins. Sequestration of essential proteins and subsequent growth arrest was tested using yeast strains with galactose-inducible control of Pex5p expression. Strains shown were modified so that one of four essential proteins (Cdc14p, Cdc28p, Tys1p or Spc42p) was individually fused to either ePTS1 or naïve PTS1. Control strains with a permanently defective peroxisomal import system (*pex14*Δ) were also included. Strains were spotted onto agar plates with galactose to induce Pex5p expression and grown for 48 h. WT, wild type.

As a final test of ePTS1’s utility, we examined the tag’s modularity using an inducible sequestration assay in which the native *pex5* promoter was replaced with the galactose-inducible *gal1* promoter. Upon galactose-induction of Pex5p and thus peroxisomal import, we observed that RFP-ePTS1 was completely compartmentalized in the peroxisome after only 1 h in this strain background (Supplementary Fig. 8). We fused the PTS1 or ePTS1 tag to one of four essential genes—*cdc14*, *cdc28*, *tys1* and *spc42*—expecting that sequestration of these proteins in the peroxisome would prevent growth. Indeed, we observed that all four ePTS1-tagged constructs yielded strains that were unable to grow under galactose induction (Fig. 4). In contrast, the canonical PTS1 tag arrested growth of only two of four strains, suggesting that Cdc14p-SKL and Cdc28p-SKL were inadequately compartmentalized. We also tested these constructs with *pex14*Δ strains, which are missing a key component of the import pore complex necessary for peroxisomal protein import. As expected, all *pex14*Δ strains grew on galactose-containing media, indicating that the growth-arrest effects are import dependent and not an artefact of Pex5p binding. These results demonstrate that the ePTS1 tag maintains its rapid import properties when fused to a variety of cargo proteins.

### Small metabolites freely traverse the peroxisomal membrane

**Figure 5:**
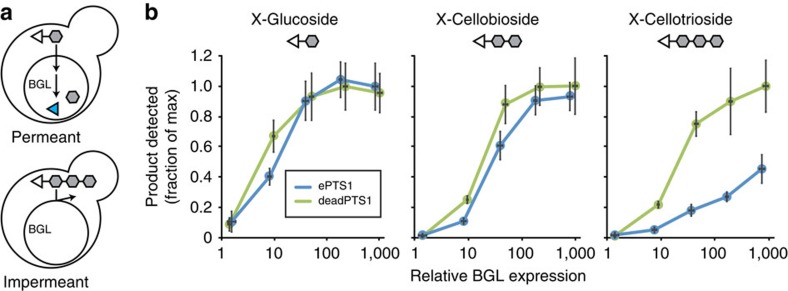
Oligosaccharide-based assay reveals size-dependent permeability of the peroxisomal membrane. (**a**) Yeast cells expressing a peroxisomally localized β-glucosidase (BGL) were fed 5-bromo-4-chloro-indoxyl (X) dye molecules (triangles) conjugated to glucose chains of increasing length (hexagons). BGL can cleave the dye-sugar conjugates, releasing the fluorescent dye molecule (blue triangle), but only if those conjugates are able to cross the peroxisomal membrane to access the BGL. Impermeant substrates should be unable to access the BGL and thus should generate no fluorescence. (**b**) Relative fluorescent product release in yeast strains expressing increasing amounts of peroxisomal (blue lines) or cytosolic (green lines) BGL. Dye–sugar reactions involving X-glucoside (*M*_w_=408.63 Da), X-cellobioside (*M*_w_=570.78 Da) and X-cellotrioside (*M*_w_=732.93 Da) are described in detail in Supplementary Fig. 9. Error bars are the mean±s.d. of six biological replicates.

Having improved our control over protein targeting, we turned to metabolite transport and applied our new tools to the challenges of measuring *in vivo* transport through subcellular compartments. Using β-glucosidase (BGL), a promiscuous enzyme from *Neurospora crassa*^34^ that accepts a wide variety of glycosides for hydrolysis, we designed a novel *in vivo* polymer exclusion assay to measure peroxisomal permeability. We reasoned that if the previously hypothesized^23^ aqueous pore were indeed present on the peroxisomal membrane, peroxisomally localized BGL would have activity on small substrates but not larger ones (Fig. 5a). We selected three structurally similar, size-diverse substrates for testing, all of which are oligosaccharides conjugated to a dye molecule: X-glucoside (molecular weight *M*_w_=409 Da), X-cellobioside (*M*_w_=571 Da) and X-cellotrioside (*M*_w_=733 Da), where ‘X’ represents 5-bromo-4-chloro-3-indolyl (Supplementary Fig. 9). When these substrates are hydrolyzed by BGL, they release 5-bromo-4-chloro-indoxyl, a fluorescent molecule that spontaneously oxidizes into the blue pigment 5,5′-dibromo-4,4′-dichloro-indigo. Because the substrates are hydrophilic, they cannot cross phospholipid membranes—including the plasma membrane—without the aid of a protein transporter. To import the substrates into the cytosol, we expressed the cellobiose transporter, CDT1, from *N. crassa*^34^, which recognized all three substrates (Supplementary Fig. 10). Once again, we used a set of constitutive promoters to titrate the expression of BGL, and created cytosolic controls with a deadPTS1 tag featuring a C-terminal leucine to threonine mutation. We also augmented BGL by fusion to VioE and YFP, which enabled us to confirm consistent expression via YFP fluorescence, and verify peroxisomal import via VioE activity measurement. When VioE-YFP-BGL-ePTS1 or -deadPTS1 were expressed in strains with cytosolic VioA and VioB, we observed a decrease in VioE activity upon ePTS1 tagging, confirming BGL was contained in the peroxisome (Supplementary Fig. 11).

We then fed the X-substrates to our CDT1- and BGL-expressing strains and compared the degree to which each substrate was hydrolyzed in strains with cytosolic or peroxisomal BGL (Fig. 5b). For the two smallest substrates, X-glucoside and X-cellobioside, we observed little to no effect of peroxisomal BGL import on hydrolysis. However, a significant sequestration effect was observed with the largest substrate, X-cellotrioside, with a 77% reduction in hydrolysis upon peroxisomal import at moderate pRPL18B levels of BGL. This indicates that peroxisomal transport impeded hydrolysis of the larger metabolite but not smaller ones, and the size cutoff matched well with *in vitro* polymer exclusion experiments on purified peroxisomes^26^ (Supplementary Fig. 12).

**Figure 6:**
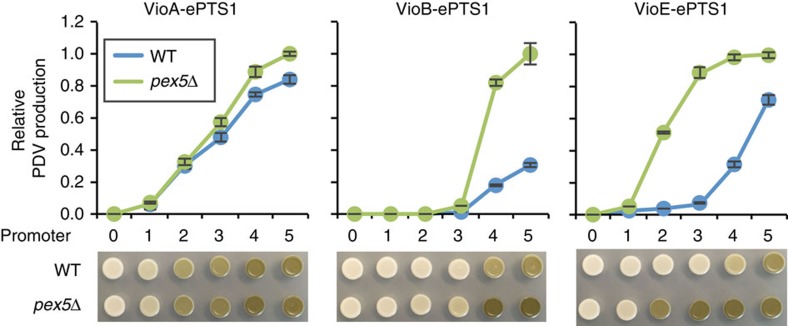
Characterization of peroxisomal permeability in the PDV pathway. PDV was extracted from yeast strains expressing an ePTS1-tagged version of VioA, VioB or VioE together with untagged versions of the remaining two enzymes. Strains were constructed in either a wild-type (WT; blue lines) or *pex5*Δ (green lines) background, with the ePTS1-tagged enzyme being expressed under a variety of promoters, numbered in rank order of expression strength: 0=no gene present, 1=pREV1, 2=pRNR2, 3=pRPL18B, 4=pTEF1, 5=pTDH3. Error bars represent the mean±s.d. of four biological replicates. The extraction data are accompanied by spots showing the yeast after 48 h growth (bottom).

Given these findings, we were interested in the effect that nonspecific permeability would have on our ability to sequester the PDV pathway. We fused the ePTS1 tag directly to the C-terminus of each enzyme and assayed activity in wild-type and *pex5*Δ strains with cytosolic expression of the other two pathway enzymes. Across a range of enzyme expression levels, we observed a considerable drop in PDV production when either VioB or VioE was targeted to the peroxisome, but not VioA (Fig. 6). The effect of VioB sequestration in the peroxisome peaked at a 4.5-fold drop in activity (at the pTEF1 promoter level), whereas sequestration of VioE achieved 13-fold reduced activity (at the pRNR2 and pRPL18B promoter levels). This relative difference in sequestration performance may be due to less efficient import of the bulky (111 kDa) VioB or directional differences in IPA imine dimer flux across the peroxisomal membrane. As the volume of peroxisomes is roughly one hundredth that of the cytosol^31^, it would be expected that the gradient driving IPA imine dimer across the peroxisomal membrane is up to 100-fold greater when VioB is in the peroxisome. Although VioB sequestration was not as effective as VioE sequestration, the two enzymes’ shared intermediate, IPA imine dimer, appears to be mostly peroxisome-impermeant.

To verify the null effect of peroxisomal targeting on VioA, we again turned to the VioE-fusion sequestration assay. As illustrated in Supplementary Fig. 13, a VioE–VioA fusion was expressed either in a system designed to test VioE activity by expressing VioA and VioB cytosolically or in a VioA-monitoring strain where VioB and VioE were expressed cytosolically, with *pex5*Δ controls for each. We found that the VioE–VioA fusion lost VioE activity when targeted to the peroxisome, demonstrating efficient peroxisomal import (Supplementary Fig. 13b,c). In contrast, the VioA portion of the fusion remained active irrespective of peroxisomal targeting. These results provide *in vivo* evidence that only VioA remains active as part of the PDV pathway while peroxisomally sequestered, further suggesting that both VioA’s substrate (tryptophan) and product (IPA imine) can cross the peroxisomal membrane efficiently, but not VioB/VioE’s IPA imine dimer.

### PDV production can be compartmentalized in certain regimes

Nonspecific permeability to small intermediates poses a problem for the isolation of pathways in the peroxisome. Although our enzyme sequestration assays could prove useful for identifying the source of this permeability, our initial efforts to do so have proven unsuccessful. Even so, the last two steps in the PDV pathway, VioB and VioE, share an intermediate, IPA imine dimer, which does not appear to efficiently cross the native peroxisomal membrane. IPA imine dimer can also spontaneously form the dead-end side product chromopyrrolic acid (CPA; Supplementary Fig. 1). We hypothesized that, by compartmentalizing VioB and VioE in the peroxisome, we could demonstrate both enzymes remain functional in the peroxisome, and potentially even reduce CPA formation by channelling IPA imine dimer to VioE.

First, we had to account for the import of an additional enzyme by measuring how much displacement occurs when multiple heterologous cargo proteins compete for peroxisomal import. To do this, we expressed a constant amount of peroxisomally targeted VioE in a strain that overexpressed cytosolic VioA and VioB. We then investigated how much of a competitor protein, RFP-ePTS1, must be expressed to competitively displace VioE from the peroxisomal import system (Supplementary Fig. 14a). With no competitor expressed, VioE was efficiently sequestered in the peroxisome, and the strain produced 95% less PDV than its *pex5*Δ control (Supplementary Fig. 14b,c). Expression of the competitor had negligible effects on VioE-targeting and PDV production even up to the strongest single promoter, pTDH3, where a drop from 95 to 85% VioE sequestration was observed. Only when multiple copies of pTDH3 were used to express RFP-ePTS1 was VioE-ePTS1 effectively displaced, suggesting that the peroxisomal import machinery possesses plenty of excess capacity.

**Figure 7:**
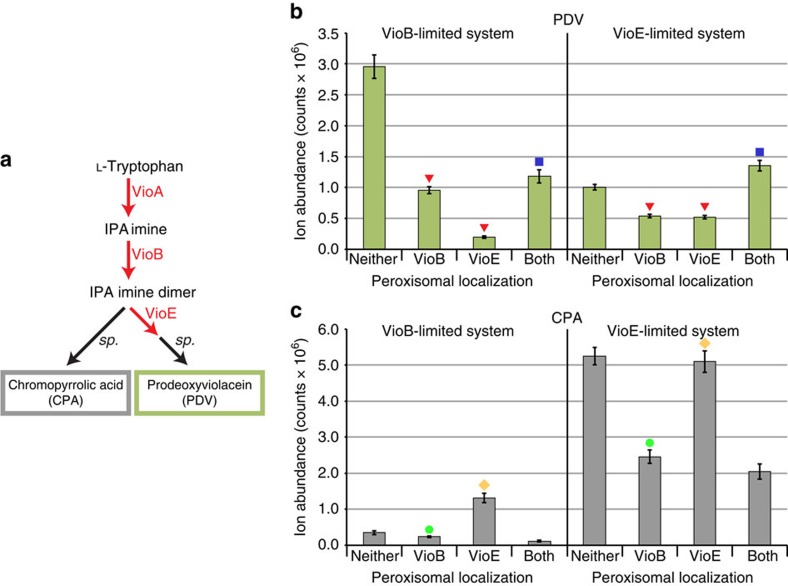
Co-localizing PDV pathway enzymes in the peroxisome reduces by-product CPA in all cases and also increases PDV production in a VioE-limited regime. (**a**) The PDV pathway, now including chromopyrrolic acid (CPA), the colourless spontaneous by-product that spontaneously forms from IPA imine dimer in the absence of VioE activity. (**b**,**c**) Bars show amounts of PDV or CPA detected in yeast expressing VioB and VioE in the cytosol or peroxisome, with error bars showing the mean±s.d. of six biological replicates. Two expression regimes of VioB and VioE are tested: a VioB-limited regime with less VioB and more VioE, and a VioE-limited regime with more VioB and less VioE. (**b**) Upon separation of VioB and VioE into different compartments, PDV production consistently decreases (red ▾) relative to both enzymes together in the cytosol. However, when VioB and VioE are together in the peroxisome (blue ▪), the change in PDV production relative to cytosolic localization depends on whether excess VioB is present, with excess VioB allowing a net increase in PDV. (**c**) CPA formation generally decreases upon VioB import into the peroxisome (green ●), but increases or stays the same upon VioE import into the peroxisome (gold ♦).

Having established that the peroxisomal import machinery had sufficient capacity for both proteins, we moved to testing the simultaneous compartmentalization of VioB and VioE. In a strain expressing cytosolic VioA, we expressed VioB and VioE each with an ePTS1 tag for peroxisomal compartmentalization or no tag for cytosolic targeting. We used both wild-type (Fig. 7) and *pex5*Δ (Supplementary Fig. 15) cells to control for any import-independent differences in enzyme activity because of fusion of the ePTS1 tag. We were particularly interested in whether peroxisomal targeting improves PDV production when one or more enzyme partners are scarce to highlight the effects of locally concentrating enzymes and intermediates by compartmentalization. We selected two different expression regimes to test: (i) a VioB-limited regime where substrate channelling is less likely to be observed because there is plenty of VioE available to convert IPA imine dimer (pRPL18B-VioB/pRNR2-VioE) and (ii) a VioE-limited regime where substrate channelling effects should be most visible (pTEF1-VioB/pREV1-VioE).

As enzymes are targeted to the peroxisome, three trends in PDV production appear: when VioB and VioE are in separate compartments, less PDV is produced; VioB loses some of its activity upon import; and when excess VioB is provided, PDV production actually increases when the pathway is moved from the cytosol to the peroxisome. In both regimes, strains with VioB and VioE directed to the same compartment made more PDV than strains in which the enzymes were split between the peroxisome and cytosol (Fig. 7b), suggesting that both enzymes remained active and PDV was produced in the peroxisome. However, the effect of moving both enzymes from the cytosol to the peroxisome was quite different in the two regimes. In the VioB-limited expression regime, PDV production was 60% lower when VioB and VioE were compartmentalized in the peroxisome (Fig. 7b). In contrast, in the VioE-limited regime, we observed 35% greater PDV production when VioB and VioE moved from the cytosol to the peroxisome (Fig. 7b).

To examine the disparity between these regimes, we measured accumulation of the off-pathway product CPA, which forms spontaneously from the product of VioB in the absence of VioE (Fig. 7a). CPA should accumulate when VioB’s product cannot access VioE, such as when VioB and VioE are split between the peroxisome and cytosol. Indeed, we observed the expected increase in CPA when VioE was sequestered in the peroxisome in the VioB-limited regime (Fig. 7c). The VioE-limited regime showed little change in CPA, likely because of reduced pathway draw from VioE (Fig. 7c). However, peroxisomally sequestered VioB generated 33% or 53% less CPA in the VioB- and VioE-limited regimes, respectively. The consistent decrease in the CPA product from VioB upon peroxisomal localization suggests that VioB has reduced activity when imported into the peroxisome. There are several explanations for this behaviour, including an unfavourable lumenal environment due to pH^35^,^36^ or other factors, or that VioB itself is being imported in a nonfunctional state. It is possible that ePTS1-based import outpaces haeme loading of VioB, as peroxisomal import can occur before cargo folding^37^. Furthermore, it is thought that free haeme (617 Da) is unavailable in the peroxisome^38^, so apo-VioB would be unable to acquire haeme after import.

Even with the apparent decline in the specific activity of VioB in the peroxisome, if excess VioB is supplied as in the VioE-limited regime, PDV production increases by 35% upon import of both enzymes (Fig. 7b). We suspect this increase is not due to protection of VioE from degradation, as a slight decrease in YFP fluorescence was observed when VioE-YFP was successfully imported into the peroxisome (Fig. 2b). Instead, we hypothesize that either the lumenal environment is favourable for VioE activity or substrate channelling is occurring. Substrate channelling could be created by co-localizing the source (VioB) and sink (VioE) of IPA imine dimer, thus reducing the accumulation of IPA imine dimer and reducing the formation of CPA by-product. Under this specific expression regime, we found that wasted flux can be decreased and desirable production increased by co-localization of VioB and VioE in the peroxisome.

## Discussion

Engineering pathway compartmentalization requires precise control over both protein and metabolite localization, but the tools for measuring and manipulating these traits in peroxisomes are lacking. We report the development of novel *in vivo* assays for analysing protein and metabolite import into the peroxisome as well as the identification of an efficient, modular targeting tag to direct heterologous cargo to the yeast peroxisome. These tools offered new insights into basic peroxisome biology and allowed us to establish key design principles for repurposing the peroxisome to compartmentalize engineered metabolic pathways.

A fundamental requirement of a synthetic organelle is the ability to import protein effectively and minimize cytosolic activity of cargo proteins before import. We find the yeast peroxisome to be highly efficient in this regard, with the native peroxisomal import machinery able to handle import of cargo driven by multiple copies of the high-strength promoter, pTDH3. As a single copy of pTDH3 can drive production of ∼4% of the cell’s total protein^39^, import capacity should not be a problem for most protein-based applications. Even so, further enhancements should be achievable by overexpressing peroxisome biogenesis genes, removing endogenous peroxisomal cargo, or using hosts with naturally voluminous peroxisomes, such as *Pichia pastoris*^10^. Speed of protein import is also satisfactory, as we found that an enhanced PTS1 (ePTS1) tag bearing a linker with several basic residues was able to effect rapid peroxisomal import for a wide variety of proteins. Cargo proteins were compartmentalized at rates that outpaced TEV protease in a competition assay, arrested growth in essential protein sequestration assays and cleared the cytosol of fluorescent protein within an hour of induction. Although the ePTS1 tag appears to be modular, we have developed a novel assay that can aid in high-throughput measurement and optimization of an individual protein’s import efficiency. By fusing the enzyme VioE to a protein of interest, the degree of sequestration of the fusion in the peroxisome can be determined by loss of VioE’s product, the pigment PDV. With the native protein import machinery capacious, rapid and versatile, and several tools available to help make further improvements, we moved on to the next challenge: permeability.

A second fundamental requirement of a synthetic organelle is control over metabolite transport in order to import substrates, contain intermediates and exclude cross-reactive metabolites. Our initial efforts to sequester heterologous enzymes suggested that the peroxisomal membrane allows free diffusion of some, but not all small hydrophilic metabolites consistent with the previously hypothesized peroxisomal aqueous pore with a molecular weight cutoff of 300–400 Da (ref. 23). To get a better estimate of pore size, we developed a BGL-based assay to generate the first *in vivo* polymer exclusion measurements for peroxisomal permeability, estimating the pore radius between 0.57 and 0.65 nm. This value was consistent with *in vitro* polymer exclusion measurements^26^ and suggests the 2–4 nm permeability states estimated using reconstituted peroxisomal protein and ion conductance^16^ are unlikely to be encountered in metabolic engineering applications. Although our estimate will be predictive for stable molecules, reactivity and molecular lifetime also play a role, as the near-diffusion-limited enzyme catalase shows latency even though its substrate, hydrogen peroxide (34 Da), is thought to easily cross the peroxisomal membrane^24^. Similar effects might explain our ability to compartmentalize the reactive intermediate of the PDV pathway, IPA imine dimer. For pathways with stable intermediates, however, the current threshold of peroxisomal permeability poses a problem. Engineered pathways frequently feature small intermediates that can be compartmentalized only if peroxisomal membrane permeability is reduced. We expect this permeability for hydrophilic molecules is due to one or more membrane proteins rather than the lipid membrane itself. Pxmp2 has been identified as a channel-forming peroxisomal membrane protein^40^ in mammalian cells, but no equivalent channel has been confirmed in yeast. It is also possible that the protein importomer, which opens to a reported radius of 4.5 nm when importing large cargo^16^, could be the culprit. Testing this hypothesis will likely require inactivation of the importomer and development of an alternative route for directing cargo to the peroxisomal lumen, a considerable challenge. Thus, peroxisomal permeability remains an open challenge that places hard restrictions on which pathways can be effectively sequestered.

With these protein import tools and permeability rules, we were able to produce the first rigorous demonstration of a heterologous pathway compartmentalized in the peroxisome with a trapped intermediate. The model pathway consisted of VioB and VioE, which share a peroxisome-impermeant intermediate, IPA imine dimer, generated from a permeant substrate, IPA imine. Both VioB and VioE were active in the peroxisomal lumen, but VioB appeared to halve in activity upon import, whereas activity of VioE increased. With excess VioB, we found that peroxisomal import led to a 35% increase in production of PDV and a 61% reduction in the off-pathway by-product CPA. VioB’s loss of activity highlights the need for further modification of the peroxisome’s lumenal environment to maintain or enhance the activity of individual enzymes, especially cofactor-dependent enzymes such as VioB. On the other hand, VioE’s gain in activity highlights the potential for the peroxisome to enhance production, potentially by concentrating scarce enzymes and intermediates to achieve substrate channelling. Together, these results provide a glimpse into the rewards a synthetic organelle can offer and the future cost of constructing that organelle.

With its rapid, versatile import system and disposable native function, the peroxisome holds great promise and is already suitable for protein import-only applications, but several challenges stand in the way of unlocking its full potential. Reductions in small-molecule permeability and accommodations to maintain enzyme activity will be important next steps, with an eye towards the final goal of modifying the chemical environment of the peroxisomal lumen (for example, pH, redox state, cofactor balance) to allow catalysis of reactions that would be infeasible in the cytosol. Achievement of these long-term goals could one day provide unprecedented control over the chemistry that can be performed in the cell.

## Methods

### Strains and growth media

The base *S. cerevisiae* strain for all experiments in this article was BY4741 (*MATa his3Δ1 leu2Δ0 met15Δ0 ura3Δ0*). Base strain BY4741 and BY4741 *pex5*Δ (Strain 1) were both ordered from Open Biosystems—GE Dharmacon. Wild-type yeast cultures were grown in YPD (10 g l^−1^ bacto yeast extract; 20 g l^−1^ bacto peptone; 20 g l^−1^ dextrose). Selection of auxotrophic markers (URA3 or LEU2) was performed in synthetic complete media (6.7 g l^−1^ Difco Yeast Nitrogen Base without amino acids (Spectrum Chemical); 2 g l^−1^ Drop-out Mix Synthetic Minus appropriate amino acids, without Yeast Nitrogen Base (US Biological); 20 g l^−1^ Dextrose). All strains constructed for this work are listed in the Supplementary Table 2.

Golden gate assembly reactions were transformed in chemically competent *Escherichia coli* prepared from strain TG1 (Lucigen). Linker libraries were transformed in TransforMax EPI300 (Epicentre) electrocompetent *E. coli*. Transformed cells were selected on Lysogeny Broth containing the antibiotics ampicillin or kanamycin. Sequences of plasmids produced for this work are supplied in a zip archive (Supplementary Data 1).

### Yeast strain construction

Yeast expression vectors were built using Golden Gate Assembly as described in the YTK system^30^. All plasmids were designed for direct integration into the yeast genome via homologous recombination at the URA3 or LEU2 or HIS3 locus. Five hundred nanograms of plasmid were linearized by digestion with *Not*I and transformed directly into yeast using a standard lithium acetate transformation^41^. Cells were plated onto dropout plates corresponding to the auxotrophic marker present in the integration cassette. Replicate colonies were picked directly from this transformation plate for further analysis. The genomic modifications in Strains 54 and 56–71 were generated using CRISPR/Cas9, in a manner outlined in the yeast toolkit^30^. Cells were co-transformed with a Cas9+sgRNA expression vector and a repair DNA. Transformants were grown on uracil-selective media to ensure maintenance of the plasmid until the desired genomic modification could be confirmed by sequencing, at which point the cells were transferred to 5-fluoroorotic acid (1 g l^−1^) plates to encourage clearance of the plasmid. Once the plasmid clearance was verified by PCR for loss of the URA3 marker, the strains were ready for use. Sequences of the modified loci and the linear repair DNA produced for this work are supplied in a zipped archive (Supplementary Data 1).

### Linker library construction

The six-amino-acid linker library was constructed by digesting the pRPL18B-VioE expression vector pWCD1661 with *Bam*HI and *Xho*I and inserting the annealed oligos EA03 (5′-GATCTGGTAGCDNKDNKDNKDNKDNKDNKTCCAAATTGTAAC-3′) and EA04 (5′-TCGAGTTACAATTTGGAMNHMNHMNHMNHMNHMNHGCTACCA-3′), which together contained the (DNK)_6_ region followed by the canonical PTS1 and a stop codon. Once ligated, this new plasmid library (pWCD2420L) was transformed into EPI300 *E. coli* as described above. Colonies from those transformations were pooled, miniprepped and the resulting DNA was transformed into Strain 2 and plated on synthetic complete 2% dextrose minus uracil to generate the library (Strain 11) used in Fig. 2a and Supplementary Fig. 3.

### Fluorescence microscopy

For confirmation of protein localization, strains were grown in synthetic complete media (minus prototrophic nutrients) with 2% glucose overnight, back diluted 50-fold into fresh media, and grown for an additional 6 h. Cultures were concentrated by centrifugation, washed in pH 7.4 PBS and spotted onto plain glass slides to be examined with a confocal fluorescence microscope.

Strains 9, 10 and 40–51 were examined with confocal microscopy using a CSU-X1 spinning disk confocal microscope (Yokogawa) with Leica optics including a × 100 objective and 442, 488 and 561 nm excitation lasers for cyan fluorescent protein (CFP, specifically mTurquoise2), YFP (Venus) and RFP (mKate2), respectively. Images were taken using a QuantEM 512SC EMCCD camera (Photometrics). All images were analysed using Fiji (http://fiji.sc).

### Fluorescent protein measurements

For quantification of relative amounts of fluorescent proteins, cells were pelleted and resuspended in an equal volume of PBS, pH 7.4, to remove background fluorescence. One hundred microlitres of cells were transferred to a microtitre plate and fluorescence was measured on a Tecan Safire2 or M1000 infinite plate reader. RFP (mKate2) was detected at excitation 580/5 nm and emission 617/5 nm, YFP (Venus) was detected at excitation 516/5 nm and emission 530/5 nm, and CFP (mTurquoise2) was detected at excitation 435/5 nm and emission 478/5 nm.

### Plate and spot assays for PDV production

Libraries were generated and plated as described above (Supplementary Fig. 3), whereas spots for PDV production were generated by plating 10 μl of saturated culture on synthetic complete 2% dextrose plates missing uracil. Unless otherwise noted, plates were grown for 48 h at 30 °C before pictures were taken using a consumer digital camera.

### Production of PDV

Yeast expressing the PDV pathway (Strains 12–33, 76–109, 124–134, 138–161 and 164–179) were grown overnight at 30 °C with 750 r.p.m. shaking in synthetic dextrose medium without uracil (SD-Ura). Saturated cultures were then diluted 50-fold into 500 μl of fresh SD-Ura and grown for 48 h, at which point 100 μl of culture was taken for fluorescent protein measurement. The remainder continued incubating for another 12 h (for a total of 60 h) before PDV extraction was done.

### Extraction and quantification of PDV production

Three hundred microlitres of cells were pelleted and the spent media were discarded. Cells were resuspended in 150 μl of glacial acetic acid and transferred to thin-walled PCR tubes. The tubes were then incubated at 95 °C for 15 min, mixed by inversion and incubated for another 15 min. Cell debris was removed first by centrifugation for 5 min at 4,700 r.p.m. (4,816*g* relative centrifugal force (RCF)) and subsequently by filtration of the supernatant with an Acroprep Advance 0.2 μm filter plate (Pall Corporation). One hundred microlitres of filtered extract were then transferred to either glass vials or a microtiter plate for quantification via HPLC/MS or bulk fluorescence.

Extracts were separated on an Agilent 1260 Infinity Quaternary LC System using a Zorbax SB C18 (30 × 2.1 mm^2^, 3.5 μm particle size) reversed phase column at 30 °C using a 0.5 ml min^−1^ flow rate and an established protocol^42^. Briefly, compounds were separated using a 12-min complex gradient consisting of: start at 5% B; hold at 5% B for 1.5 min; 16.9% per minute to 98% B; hold at 98% B for 2 min; 3.1% per second to 5% B; hold at 5% B for 2.5 min. Buffer A was water with 0.1% (v/v) formic acid and buffer B was acetonitrile with 0.1% formic acid. For Strains 164–179, PDV was detected with an Agilent 6520 Q-TOF mass spectrometer in positive mode at a retention time of 5.55 min and *m*/*z* [M+H]^+^ of 312.12 and CPA was detected at a retention time of 5.45 min and *m*/*z* of 386.11. For Strains 12–33 and 76–109, PDV product was detected with an Agilent diode array detector at 610 nm.

In the course of our experiments, we found that PDV production was associated with the development of a strongly fluorescent product detectable at excitation 535/5 nm and emission 585/5 nm. Although we were not able to identify the product, we were able to determine that the bulk fluorescence of 100 μl extracts measured on a Tecan M1000 microplate reader linearly corresponds to the amount of PDV detected in those extracts when separated and quantified by HPLC/MS (*R*^2^=0.997; Supplementary Fig. 4). Thus, for high-throughput applications such as the linker library (Strain 11) as well as some of our experimental validation (Strains 124–134), we chose to rely on bulk fluorescence for estimating PDV production.

### Library quantification and fitting

PDV production of the linker library was quantified fluorescently as described above, and normalized by subtracting autofluorescence from VioE-deficient cells and dividing by productivity in VioE-expressing cells with defective peroxisomal import (*pex5*Δ). Net charge was calculated and plotted against relative PDV production using Plotly (http://plot.ly) as seen in Fig. 2a. A logistic model was then devised to model charge versus relative PDV production, using the equation 
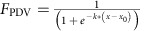
 where *F*_PDV_ is the relative amount of PDV production (ranging from 0 to 1), *x* is the net charge of the linker at physiological pH (either unweighted or weighted scores) and *k* and *x*_*0*_ are fitting parameters. The logistic curve was fit to the full data set using SigmaPlot 13 (Systat Software; Supplementary Table 1). Parameters for best fit with unweighted net charge were *k*=−1.1394 and *x*_*0*_=−0.8469 with an *R*^2^=0.5392 and SER=0.1928, whereas the parameters with net charge weighted twice as heavily for the three residues nearest the PTS1 were *k*=−0.7574 and *x*_*0*_=−1.3515 with an *R*^2^=0.6067 and SER=0.1781.

### TEV cleavage assay and western blotting

Strains 40–53 were grown to saturation in synthetic complete media (minus uracil) with 2% glucose overnight and back diluted 50-fold into fresh media. After 6 h of growth with shaking at 30 °C, confocal fluorescence microscopy was performed as described above. Relative expression levels for TEV-CFP were determined using bulk fluorescence as described above. The relative fluorescence was obtained via pixel saturation measurement using FIJI (http://fiji.sc), and relative amounts of cleaved and uncleaved protein were calculated as follows: Based on 0.051 μm^2^ average cross-sectional area per peroxisome and 0.98 peroxisomes per μm^3^ of cell volume from Tam *et al.*^31^, we calculate peroxisomes account for 0.85% of the total cell volume. Further algebraic conversion establishes the fraction of protein cleaved before import is equal to 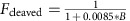, where *B* is the relative brightness of the peroxisome over cytosol.

For western blotting, 2.5 ODs of cells were pelleted, washed in water, resuspended in 200 mM NaOH and incubated at room temperature for 5 min. Cells were again pelleted, resuspended in 50 μl of polyacrylamide gel electrophoresis (PAGE) sample buffer and boiled at 95 °C for 5 min. Samples were diluted tenfold in PAGE sample buffer and 8 μl was loaded onto a NuPAGE Novex 4–12% Bis-Tris gel (Life Technologies) and run for 2.5 h at 80 V. Proteins were transferred onto polyvinylidene difluoride transfer membrane in NuPAGE transfer buffer (Life Technologies) and blocked overnight in TBST (0.05% Tween-20) with 5% milk. The membrane was washed twice with TBST for 5 min and incubated for 1 h with a rabbit polyclonal anti-RFP antibody Evrogen #AB234 (Evrogen), at a dilution of 1:5,000. After six 5-min washes in TBST, the membrane was incubated for 1 h with an horseradish peroxidase-conjugated anti-rabbit antibody (Abcam, #ab16284) at a dilution of 1:5,000. After six 5-min washes in TBST, the secondary horseradish peroxidase antibody was detected by chemiluminescence using a ChemiDoc XRS imager (Bio-Rad).

### Inducible Pex5p expression and RFP sequestration time course

The 533 bases of the region upstream of GAL1 were inserted before the start codon of PEX5 to serve as a new, inducible promoter using a CRISPR/Cas9 system as outlined above. This new *pPex5::pGal1* strain, Strain 54, was further transformed to constitutively express RFP-ePTS1, producing Strain 55. Cells were grown in synthetic complete with 2% raffinose, and in the conditions shown the cells were washed with PBS and resuspended in synthetic complete media with 2% raffinose and 1% galactose and immediately placed on a standard microscope slide with coverslip. No additional action was used to immobilize the cells. Fluorescence was periodically measured on a Zeiss Axioexplorer D1 with a × 100 DIC objective using the Texas Red 45 filter set (excitation 560/40 nm, emission 630/75 nm).

### Essential protein sequestration

Strain 54 was further modified to add an ePTS1 (GSLGRGRRSKL) or PTS1 (SKL) to the end of Cdc14p, Cdc28p, Tys1p or Spc42p while leaving the native promoter and terminator intact, resulting in Strains 56–63. These strains were then further modified by a CRISPR-mediated complete deletion of the *PEX14* ORF, resulting in Strains 64–71 with a *pPEX5::pGAL1 pex14*Δ genotype. Strains 56–71 were then propagated on YPD, and overnight cultures were washed in synthetic complete with 2% raffinose and then diluted into the same at a 1:100 dilution. After dilution, cells were allowed to grow at 30 °C, 750 r.p.m. for 2 h before 10 μl of cells were plated onto 2% agar plates containing synthetic complete with 2% raffinose and either 1% galactose or 0% galactose. Plates were incubated at 30 °C for 48 h, after which a picture was taken.

### β-Glucosidase mediated permeability measurements

Strains 112–123 expressing the cellodextrin transporter CDT1 and the fusion protein VioE-Venus-BGL-ePTS1 or -deadPTS1 (created by mutating the final residue of the ePTS1 tag to threonine) were grown overnight in YPD. Then, these cultures were diluted 1:100 into synthetic dextrose medium missing uracil and leucine, and incubated shaking at 750 r.p.m. for 14 h at 30 °C. All following work was done at room temperature (22 °C). The cells were washed with water, transferred to an anaerobic chamber, and concentrated sixfold in buffer consisting of 2 × PBS augmented with 40 mM citrate and brought to pH 3.0 with HCl and NaOH. Fifty microlitres per well of these concentrated cells were loaded into a 384-well plate, to which 50 μl of 800 μM X-substrate in water was added. This gave a final assay concentration of 400 μM X-substrate, threefold concentrated cells, 20 mM citrate, 137 mM NaCl, 2.7 mM KCl, 10 mM Na_2_HPO_4_, 1.8 mM KH_2_PO_4_, pH 3.0. The plate was then mixed by pipetting and tightly sealed to maintain the anaerobic conditions, and centrifuged twice for 30 s at 500 RCF, rotating the plate to ensure even pelleting. The plate was then transferred to a Tecan M1000 Infinite plate reader, and, 15 min after the substrates were added, fluorescence was read via the bottom, allowing a greater signal from pelleted cells.

Venus fluorescence was read at excitation 515/5 nm and emission 530/5 nm, whereas the 5-Bromo-4-chloro-3-indoxyl (BCI) fluorescence was read at excitation 410/5 nm and emission 490/5 nm. Although the hydrolysis of X-Gal and other BCI dyes is normally observed via cyan product 5,5’-dibromo-4,4’-dichloro-indigo formation and absorbance at 610 nm, the BCI monomer exists transiently and can be observed using fluorescence (Supplementary Fig. 9). Anaerobic conditions were used to prolong the lifetime of the fluorescent product, as reactions that generate the nonfluorescent cyan product are oxygen dependent. Also note that all hydrolysis experiments were done under acidic conditions (pH ∼3), as extracellular activity of BGL from lysate is suppressed and CDT1, being a proton symporter, has enhanced transport activity^43^. Strains expressing VioA and VioB were also transformed with VioE-Venus-BGL-ePTS1 and PDV was extracted and quantified fluorescently as described above (Supplementary Fig. 11).

X-Glucoside (5-bromo-4-chloro-3-indoxyl-β-D-glucopyranoside) and X-Cellobioside were obtained from Iris Biotech GmbH and X-Cellotrioside was obtained from Carbosynth US, LLC.

### Peptide affinity measurements

The C-terminal fragment (residues 295–612)^44^ of *S. cerevisiae* PEX5 was fused to an N-terminal hexahistidine tag and expressed in *E. coli* BL21 (DE3) using a pETDUET vector (Pex5C-pETDUET plasmid pBG400 was a gift of Brooke M. Gardner, UC Berkeley). Cells were induced at mid-logarithmic growth with 1 mM isopropyl-β-D-thiogalactoside and incubated for 16 h at 30 °C with 200 r.p.m. shaking. The His6-Pex5c protein was then using a HisTrap FF 5 ml nickel affinity column (GE Biosciences) and subsequently a HiTrap Q HP 5 ml anion exchange column (GE Biosciences). Protein concentration was then measured by Bradford assay, A280 nm on a Nanodrop ND1000 and SDS–PAGE densitometry against a bovine serum albumin samples of known concentration. The protein stock solution was 130 μM His6-Pex5c (5 mg ml^−1^) in a buffer of 200 mM NaCl, 25 mM HEPES, pH 7.6. 5-Carboxyfluorescein-LGRGRRSKL (5FAM-LGRGRRSKL), LGRGRRSKL and DELYKGSKL peptides were purchased from Elim Biopharmaceuticals Inc. and resuspended in water at 20, 100 and 100 mM, respectively. All peptide-binding experiments were done with an assay buffer containing 20 nM 5FAM-LGRGRRSKL in 50 mM HEPES, pH 7.4, 100 mM NaCl, 0.05% Tween-20. Experiments were equilibrated for 16 h at 4 °C before reading. All raw fluorescence and fluorescence polarization measurements were collected using a Perkin-Elmer Victor V3.5 fluorometric plate reader in 384-well small volume non-binding polystyrene plates (Greiner). Fluorescence was measured using 480 nm excitation with 30 nm bandwidth and 535 nm emission with 40 nm bandwidth; raw fluorescence was captured over 1 s, whereas fluorescence polarization was captured over 0.5 s in the L-format.

Direct binding titrations tested 6.5 μM to 0.0015, nM in twofold increments of His6-Pex5C against 20 nM of 5FAM-LGRGRRSKL. Fluorescence anisotropy was converted to fraction bound using the equation 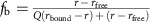 from Maynard *et al.*^19^ where *f*_b_ is the fraction of labelled peptide bound to protein, *Q* is the quantum yield ratio of bound peptide to unbound (accounting for the loss of fluorescence upon binding), *r* is the fluorescence anisotropy at a given protein concentration, *r*_bound_ is the anisotropy at maximum binding and *r*_free_ is the anisotropy with no protein bound. Key parameters found were 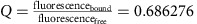, and the *K*_D_ of Pex5p C-terminal fragment binding to 5FAM-LGRGRRSKL was 355±6.38 nM.

Competition experiments started with a mixture of 375 nM Pex5c and 20 nM 5FAM-LGRGRRSKL and adding 4 mM to 3.8 nM unlabelled DELYKGSKL or 0.5 mM to 0.47 nM unlabelled LGRGRRSKL to compete off 5FAM-LGRGRRSKL from Pex5c. Fluorescence polarization data were analysed with floating values for the anisotropy of bound and unbound 5FAM-LGRGRRSKL; these values were *r*_bound_=0.2841±0.0045 and *r*_free_=0.0627±0.0025 for DELYKGSKL, and *r*_bound_=0.2993±0.0041 and *r*_free_=0.0642±0.0019 for LGRGRRSKL. Fluorescence anisotropy data were fit to curves using SigmaPlot 13 (Systat Software) and equation 17 as described in Roehrl *et al.*^45^.

## Additional information

**How to cite this article:** DeLoache, W. C. *et al.* Towards repurposing the yeast peroxisome for compartmentalizing heterologous metabolic pathways. *Nat. Commun.* 7:11152 doi: 10.1038/ncomms11152 (2016).

## Supplementary Material

Supplementary Information Supplementary Figures 1-15, Supplementary Tables 1-2 and Supplementary References

Supplementary Data 1 Sequences of all DNA constructs used in this manuscript (in '.GB' file format).
