# Supplementary material for: Towards repurposing the yeast peroxisome for compartmentalizing heterologous metabolic pathways
Source: Nat Commun. 2016 Mar 30;7:11152. doi: 10.1038/ncomms11152 (PMC5476825; doi:10.1038/ncomms11152)
Supplement: Supplementary Information — Supplementary Figures 1-15, Supplementary Tables 1-2 and Supplementary References [file ncomms11152-s1.pdf]

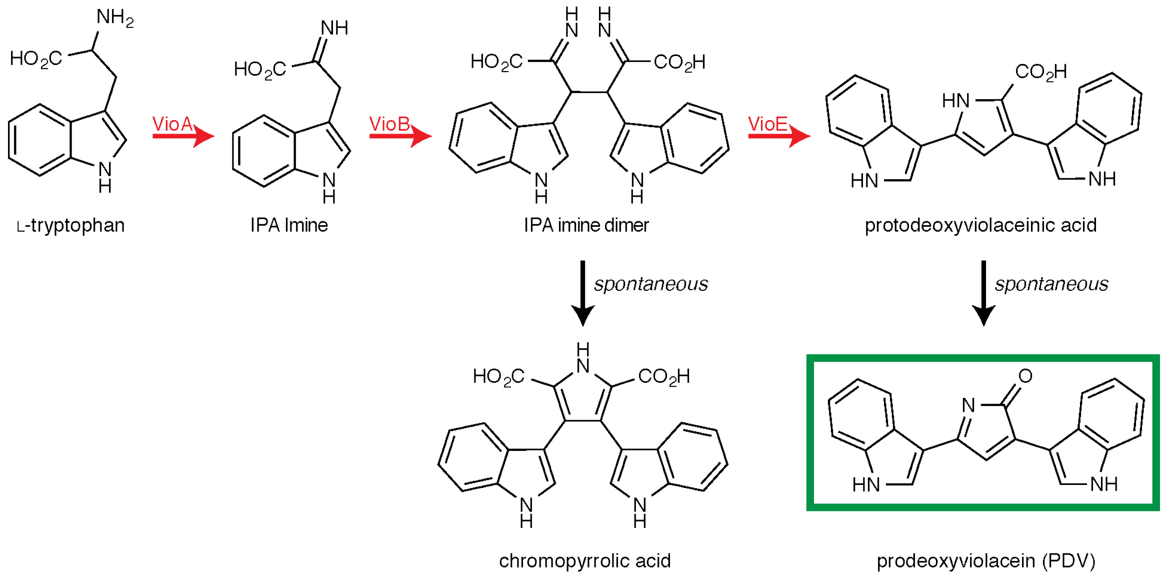

Supplementary Figure 1. The prodeoxyviolacein (PDV) pathway and its byproducts. Three enzymes, VioA, VioB, and VioE, are responsible for transforming L-tryptophan into prodeoxyviolacein (PDV). PDV alone has a visible green color; the other intemediates are colorless. The product of VioB, IPA imine dimer, is unstable and will spontaneously convert into chromopyrrolic acid (CPA) if no VioE is encountered. Red arrows denote enzymatic reactions; black arrows denote spontaneous reactions.

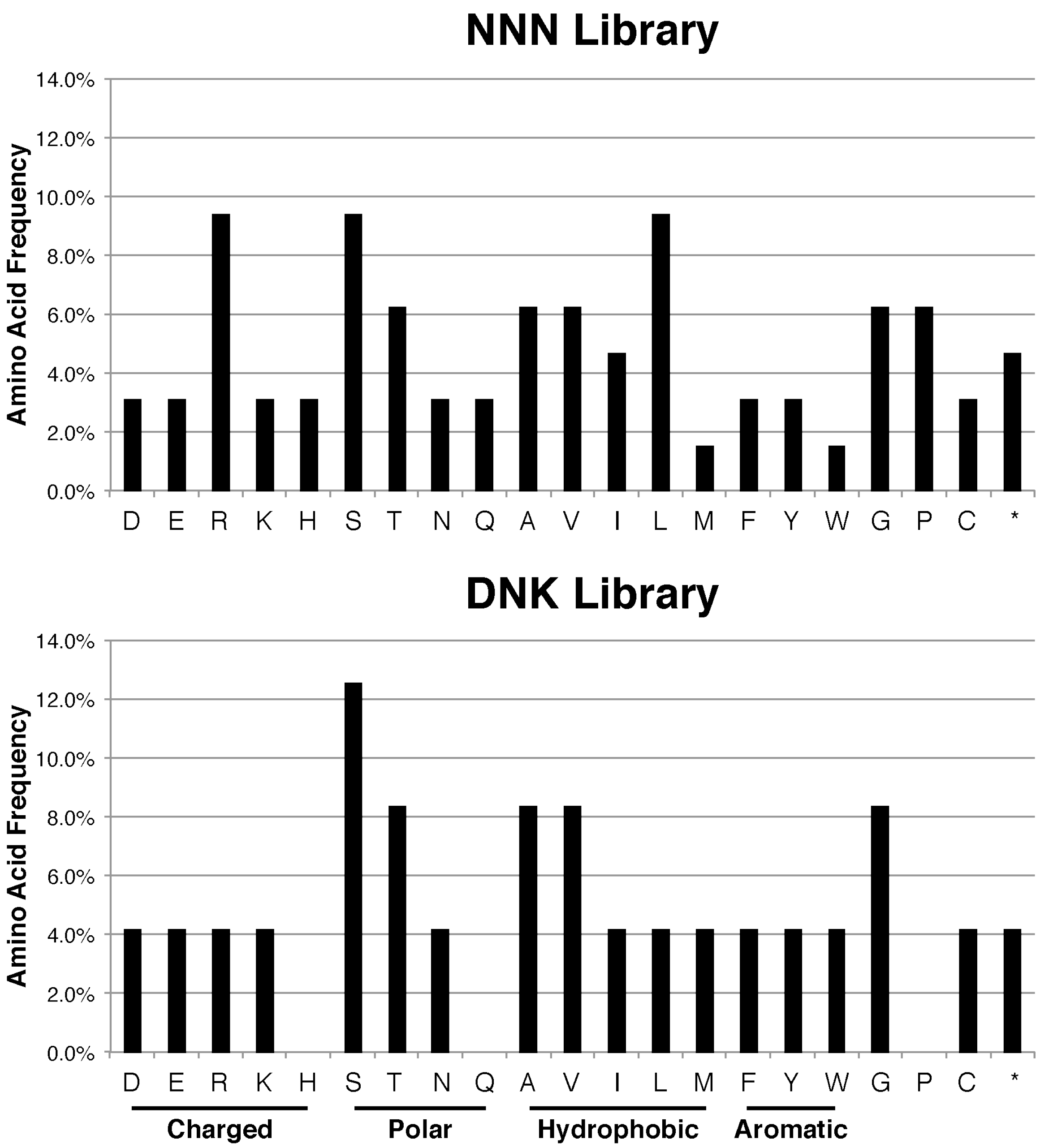

Supplementary Figure 2. Relative frequency of amino acids encoded by the degenerate codons NNN and DNK. Used in the linker library of Fig. 2A, DNK permits the following 24 different nucleotide combinations: (A,G,T)-(A,C,G,T)-(G,T). This codon provides an equal 1/12 likelihood of obtaining a positively charged basic residue (R,K) or a negatively charged acidic residue (D,E), ensuring minimal bias with regard to net charge.

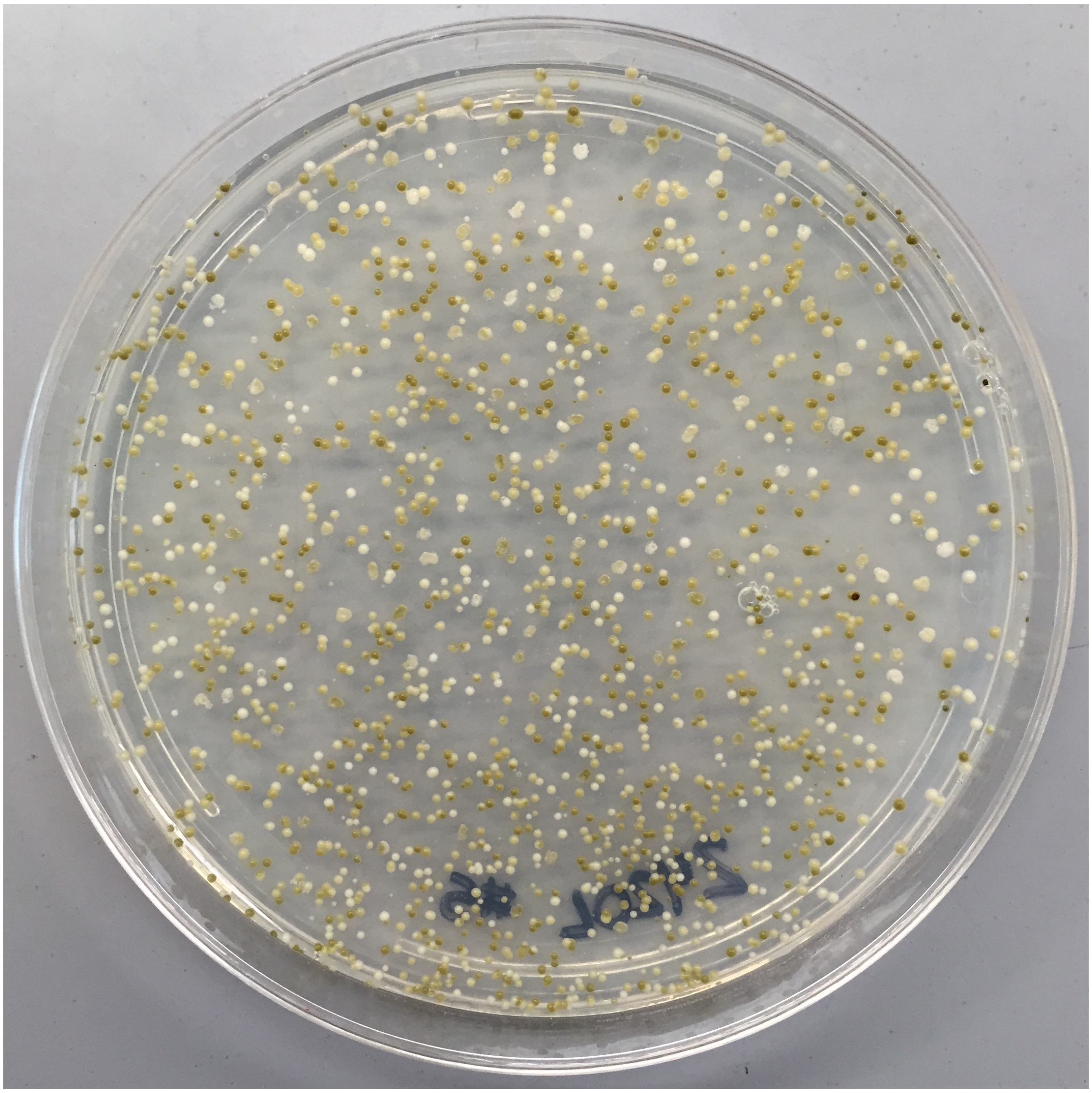

Supplementary Figure 3. Plate-based screening of linker influence on peroxisomal import using a VioE-YFP-linker-PTS1 library. Agar plate with yeast colonies coexpressing cytosolic VioA and VioB and peroxisomally-targeted VioE-YFP-linker-PTS1 after 72 hours of growth. White colonies are indicative of decreased PDV production due to peroxisomal VioE sequestration. Library members from this plate were grown and extracted to generate the data shown in Fig. 2A.

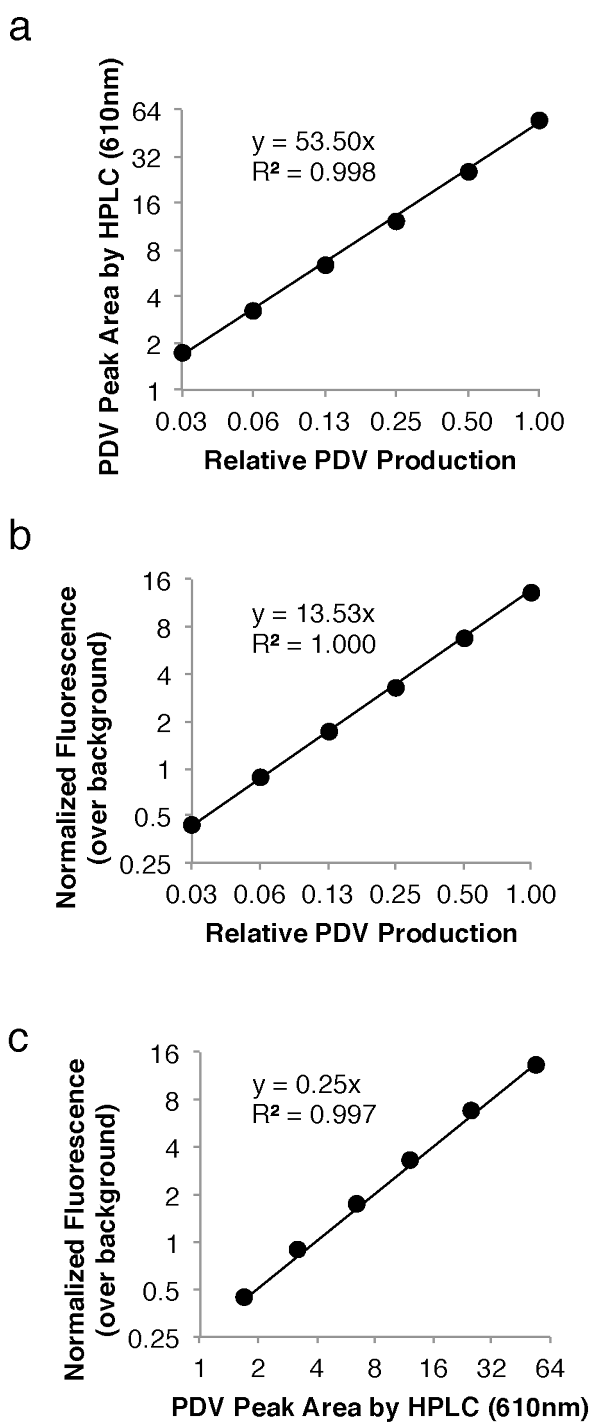

Supplementary Figure 4. HPLC absorbance and bulk fluorescence measurements of PDV show a strong linear correlation with each other and actual PDV production. Cells of high PDV-producing Strain 6 were serially diluted twofold with the cells of non PDV-producing Strain 4. Each dilution was extracted with acetic acid and measured via HPLC and bulk fluorescence to generate six-point calibration curves ranging from 1X to 0.03125X.

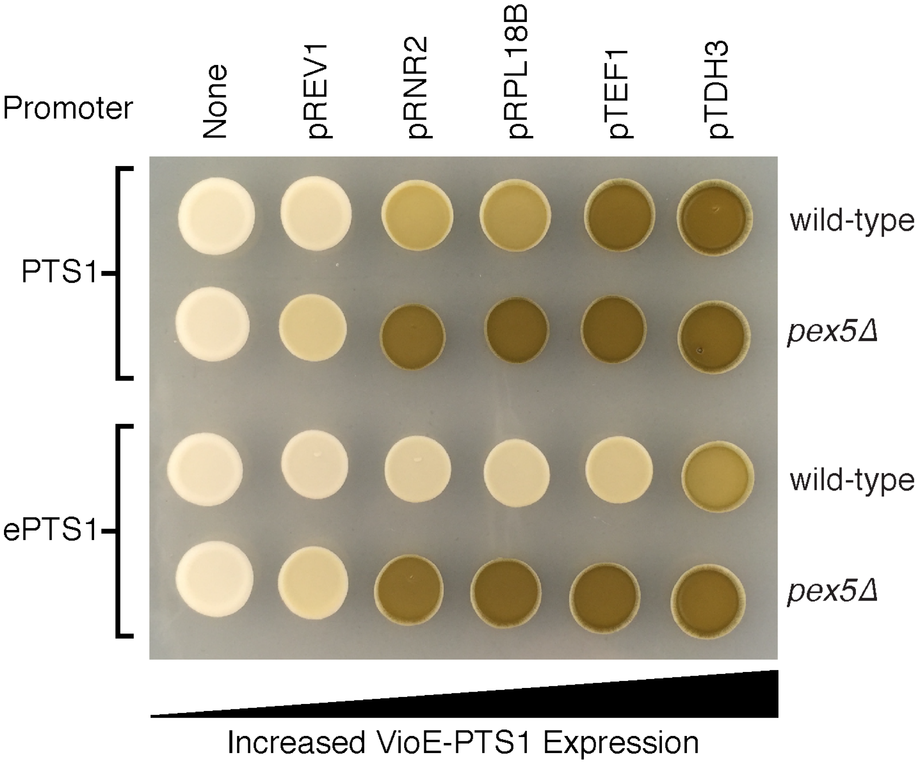

Supplementary Figure 5. Yeast spots comparing PTS1 and ePTS1 sequestration efficiency for VioE-YFP. 10 μL of saturated culture from each of the same strains used to generate Fig. 2B was spotted onto agar plates and grown for 48 hours. As in Fig. 2B, Strains 12-33 coexpressed cytosolic VioA and VioB along with VioE-YFP tagged with either PTS1 or ePTS1. Defective peroxisomal import controls (*pex5Δ*) are also shown.

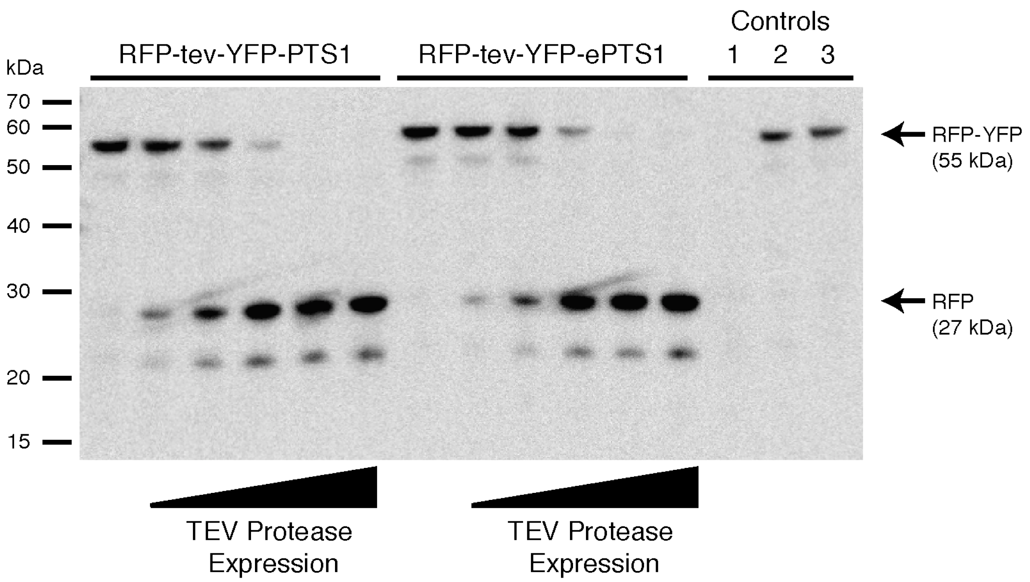

Supplementary Figure 6. Western blot analysis indicates that proteolysis of the RFP-tev-YFP fusion protein may occur in both the peroxisome and cytosol. The strains from Fig. 3, as well as strains expressing no TEV or intermediate levels of TEV (pRNR2 and pTEF1 at 4X and 45X respectively), were lysed and run on a denaturing gel, then blotted and stained with an antibody recognizing RFP. Full length (55 kDa) and proteolyzed (27 kDa) protein bands are indicated with arrows. Three controls were included to verify that TEV cleavage did not occur after cell lysis: 1) Strain 53 expressing pTDH3-TEV (140X) with no RFP-tev-YFP-ePTS1, 2) Strain 40 expressing RFP-tev-YFP-ePTS1 mixed 1:1 with Strain 52, which has only selection markers and 3) a 1:1 mixture of Strain 40 (expressing TEV protease) and Strain 53 (expressing RFP-tev-YFP-ePTS1). No RFP antibody cross-reactivity for the CFP on TEV protease was observed.

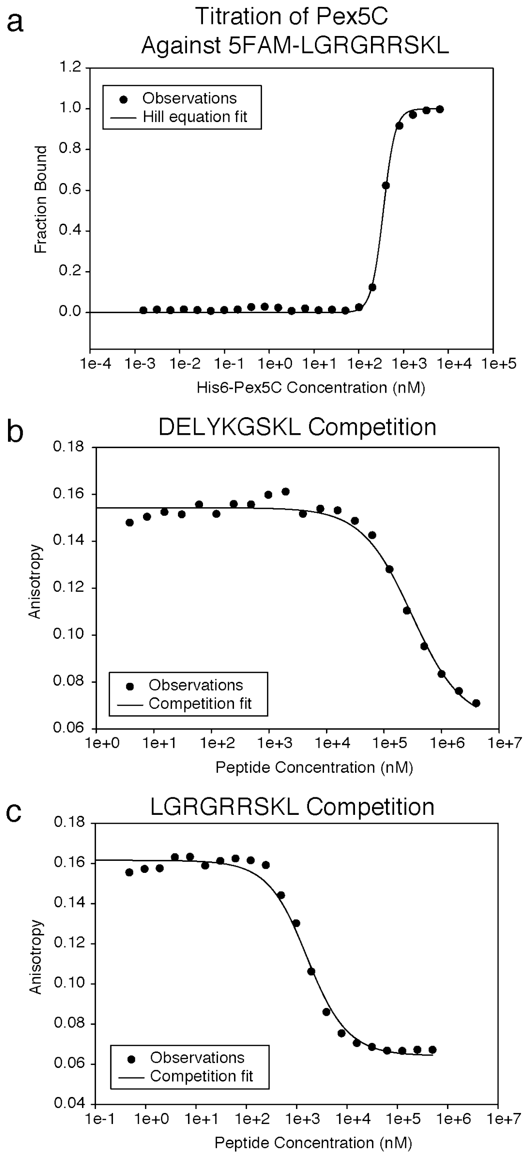

Supplementary Figure 7. Pex5p has higher affinity for ePTS1. (**a**) Direct binding assay of the C-terminal fragment of *S. cerevisiae* Pex5p against 20 nM fluorescein-labeled ePTS1 peptide 5FAM-LGRGRRSKL. Fitting this data to the Hill equation found that the K_D of Pex5p fragment binding to 5FAM-LGRGRRSKL was 355±6.38 nM with a Hill coefficient of n = 3.275±0.176. (**b,c**) Competitive binding of unlabeled PTS1 and ePTS1 peptides against 20 nM of fluorescein-labeled ePTS1 peptide. Peptides were competing for binding to 375 nM of Pex5p C-terminal fragment, and the state of the labeled ePTS1 peptide was tracked via fluorescence anisotropy. A non-cooperative model from Roerhl et al. was fit to the data, allowing estimates of relative affinities of PTS1 and ePTS1. (**b**) The unlabeled PTS1 peptide used was DELYKGSKL, reflecting the last nine amino acids of the VioE-YFP-PTS1 constructs used in Fig. 2b. The K_D found was 176000±17300nM. (**c**) The ePTS1 peptide used was LGRGRRSKL, and the K_D was 801±105 nM.

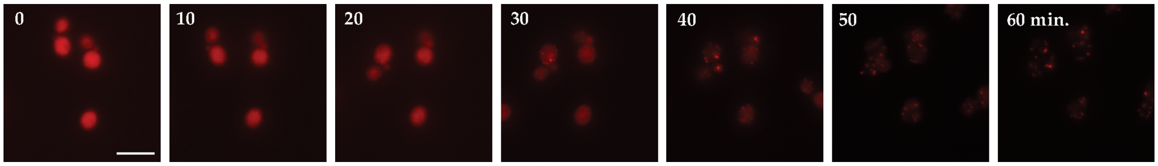

Supplementary Figure 8. Fluorescence tracking of RFP-ePTS import upon induction of Pex5p expression. At time 0, Strain 55 was transferred into synthetic complete medium with 2% raffinose and 1% galactose for induction of Pex5p expression. Fluorescence images were taken at ten-minute intervals as the newly created Pex5p shuttled RFP-ePTS1 into the peroxisomes. By 60 minutes, the cells had been virtually cleared of cytosolic RFP-ePTS1. Scale bar, 10 μM.

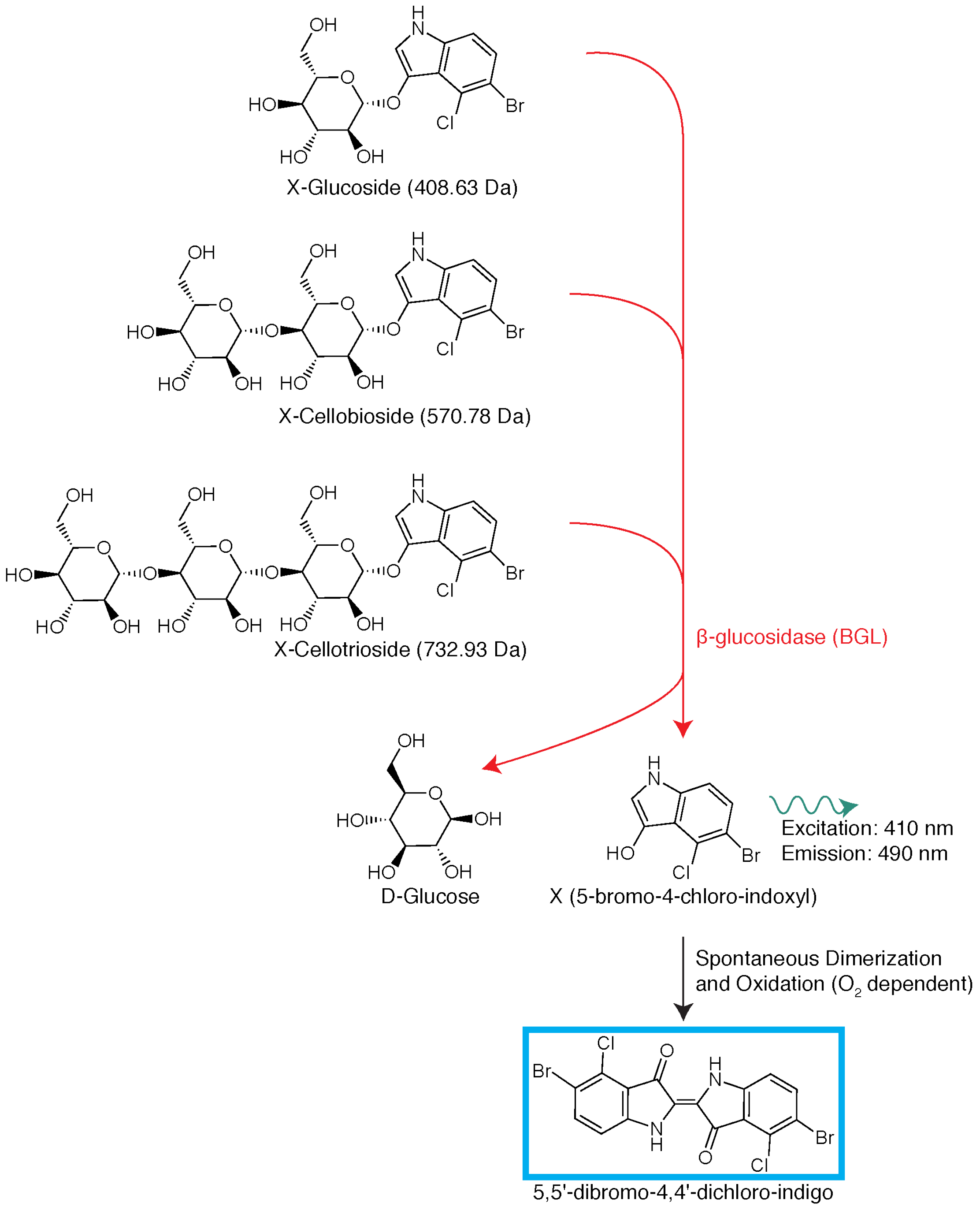

Supplementary Figure 9. Conversion of dye-substrate conjugates into fluorescent product. Upon feeding of X-dyes (also called BCI or 5-bromo-4-chloro-3-indoxyl dyes), the *N. crassa* beta-glucosidase (BGL) cleaves off sugar groups, releasing fluorescent BCI and glucose. In anaerobic environments, the spontaneous reaction of BCI to produce 5,5’-dibromo-4,4’-dichloro-indigo is diminished, extending the lifetime of the transient BCI dye and allowing fluorescent quantitation of BGL activity. In yeast, the X-dyes require expression of cellodextrin transporter CDT1 to be able to enter cells, and in the experiments shown in Fig. 5, BGL is expressed in either the peroxisome or the cytosol.

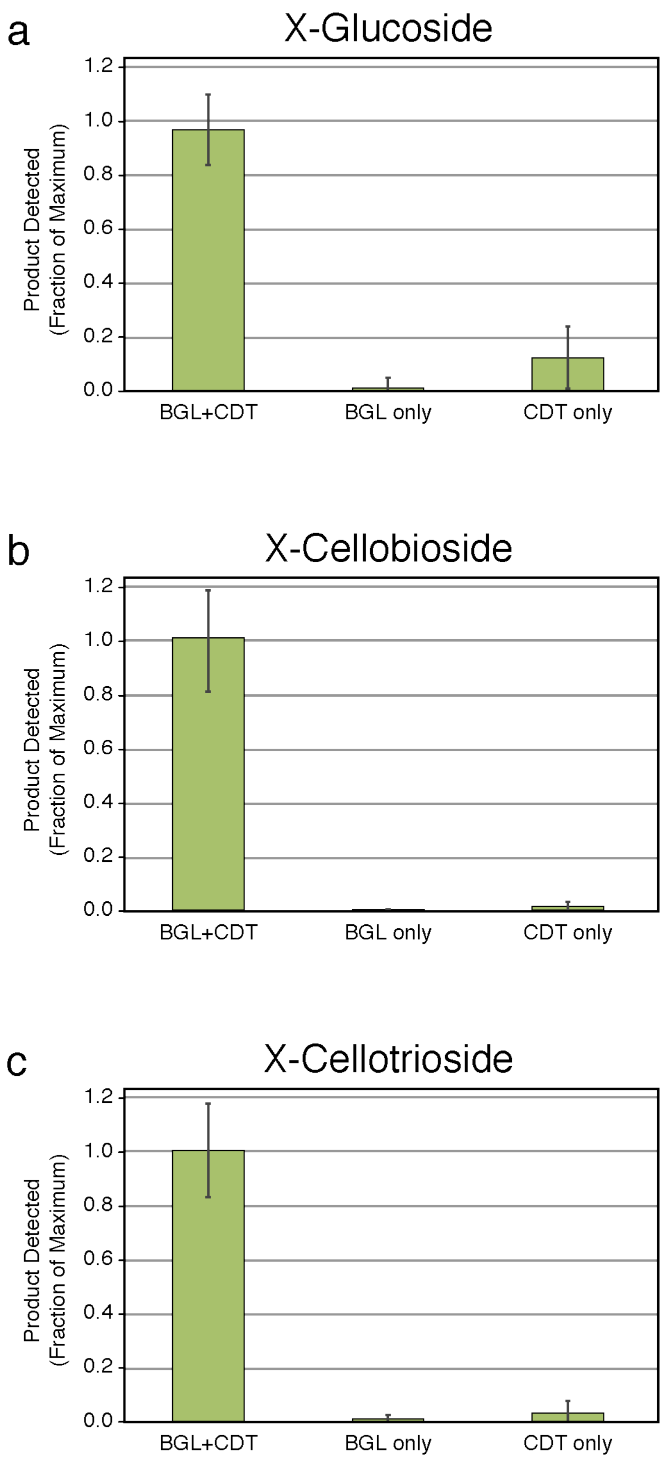

Supplementary Figure 10. Hydrolysis of X-glycosides requires coexpression of CDT1 and BGL. Strains 117, 122, and 123 express CDT1 and cytosolic BGL, BGL only, and CDT1 only, respectively. These cells were treated with X-glycosides as in Fig. 5. Error bars are the mean ± s.d. of six biological replicates.

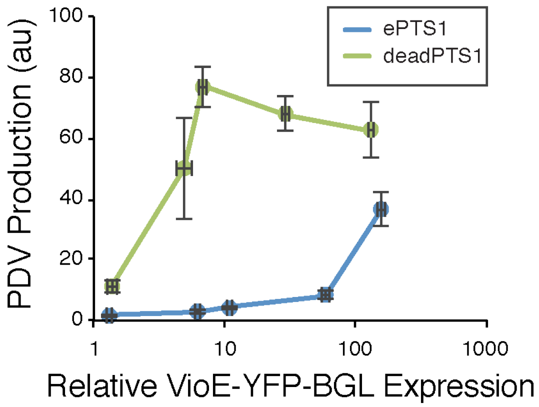

Supplementary Figure 11. Measurement of BGL import into the peroxisomes using the VioE-fusion assay. To confirm efficient BGL targeting to the peroxisome, Strain 2 coexpressing VioA and VioB cytosolically was transformed with expression cassettes for VioE-YFP-BGL-ePTS1 (the same cassettes used to make the strains shown in Fig. 5), generating Strains 124-134. These strains allowed direct testing of VioE activity by the same methods of extraction and fluorescent quantification used in Fig. 2a. The sequestration of VioE provides confirmation that the entire fusion protein is being successfully imported into the peroxisome. Error bars are the mean ± s.d. of eight biological replicates.

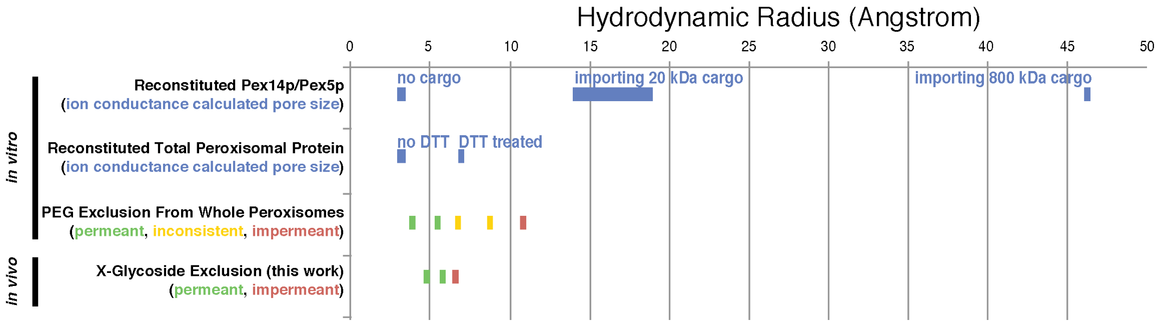

Supplementary Figure 12. Comparison of different methods of calculating peroxisomal pore size. Bars show either peroxisomal pore size estimated from an ion conductance experiment (blue) or polymers tested in exclusion experiments that consistently crossed the peroxisomal membrane (green), inconsistently crossed (yellow), or never crossed (red). Ion conductance experiments used reconstituted protein in lipid bilayers, either from total peroxisomal protein[1,2] or purified Pex14p/Pex5p[3]. Polymer exclusion experiments measured polyethylene glycol (PEG) entering whole purified peroxisomes[4] *in vitro* or X-glycosides entering peroxisomes *in vivo* as described in this work. PEG sizes tested had molecular weights of 200, 400, 600, 1000, and 1500 Da[4] and these were converted to hydrodynamic radius via previously published data[5]. X-glycosides were converted to hydrodynamic radii using experimental data for their cellodextrin counterparts[6,7]. The polymer exclusion experiments with PEG *in vitro* and X-glycosides *in vivo* agree well, placing an estimate of pore radius between 5.7 and 6.5 angstrom.

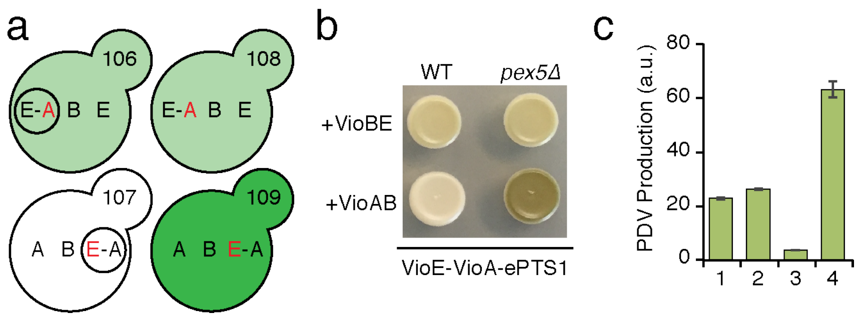

Supplementary Figure 13. Confirmation of VioA sequestration using VioE as a fusion tag for peroxisomal import measurement. (**a**) To confirm that VioA was properly localized to the peroxisome, a fusion protein VioE-VioA-ePTS1 (listed as A-E in the diagram) was expressed at pRPL18B levels along with cytosolically localized enzymes from the PDV pathway. To test the activity of VioA in the A-E fusion, VioB and VioE were coexpressed cytosolically in a wildtype (1, upper left) or *pex5Δ* strain (2, upper right). To test the activity of VioE in the A-E fusion, VioA and VioB were coexpressed cytosolically, again in a wildtype (3, lower left) or *pex5Δ* strain (4, lower right). (**b**) Yeast spots corresponding to the strains described in (**a**) after 48 hours growth. (**c**) Strains 106-109 were grown and extracted as in (**a**) and PDV measured. Error bars represent the mean ± s.d. of four biological replicates.

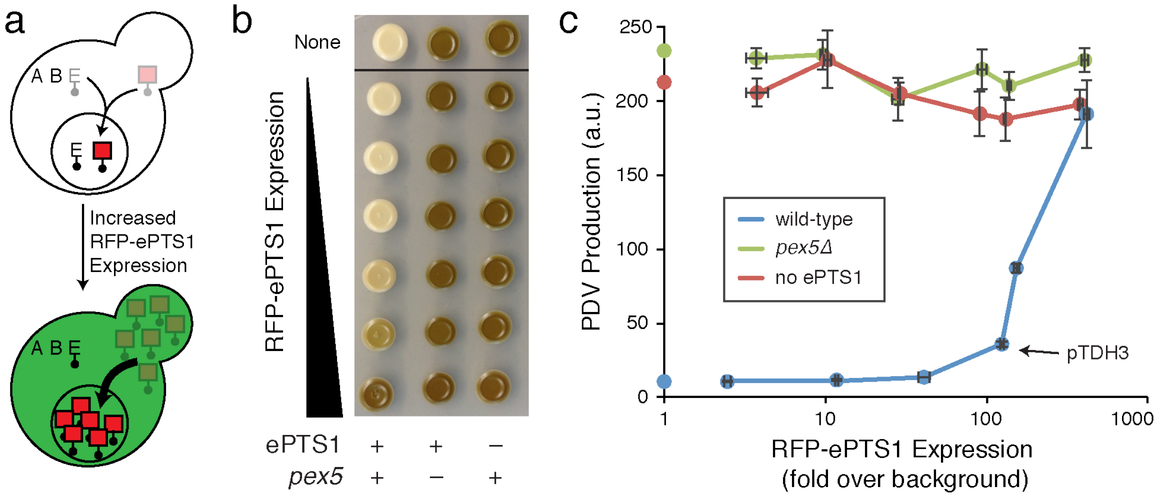

Supplementary Figure 14. Peroxisomal import can be saturated at very high levels of cargo expression. (**a**) Starting with Strains 135, 136, and 137 coexpressing cytosolic VioA, cytosolic VioB, and a moderate fixed amount of peroxisomally-targeted YFP-VioE-ePTS1, increasing amounts of RFP-ePTS1 were coexpressed in an effort to crowd VioE out of the peroxisome. If VioE was successfully prevented from entering the peroxisome by RFP cargo crowding, then increased PDV production should result as cytosolic levels of left-behind VioE rise. (**b**) PDV spot assay of Strains 138-161 showing that PDV production does indeed rise with peroxisomal cargo crowding. Controls with defective peroxisomal import (*pex5Δ*) and untagged VioE confirm the maximal level of PDV production with cytosolic VioE remains constant. (**c**) PDV extracts from the same cells shown in (**b**) to provide a more quantitative estimate of the maximum protein expression level before crowding becomes an issue. The highlighted expression level (arrow) indicates RFP being driven by a single copy of the strongest promoter in *S. cerevisiae*, pTDH3. Error bars are the mean ± s.d. of four biological replicates.

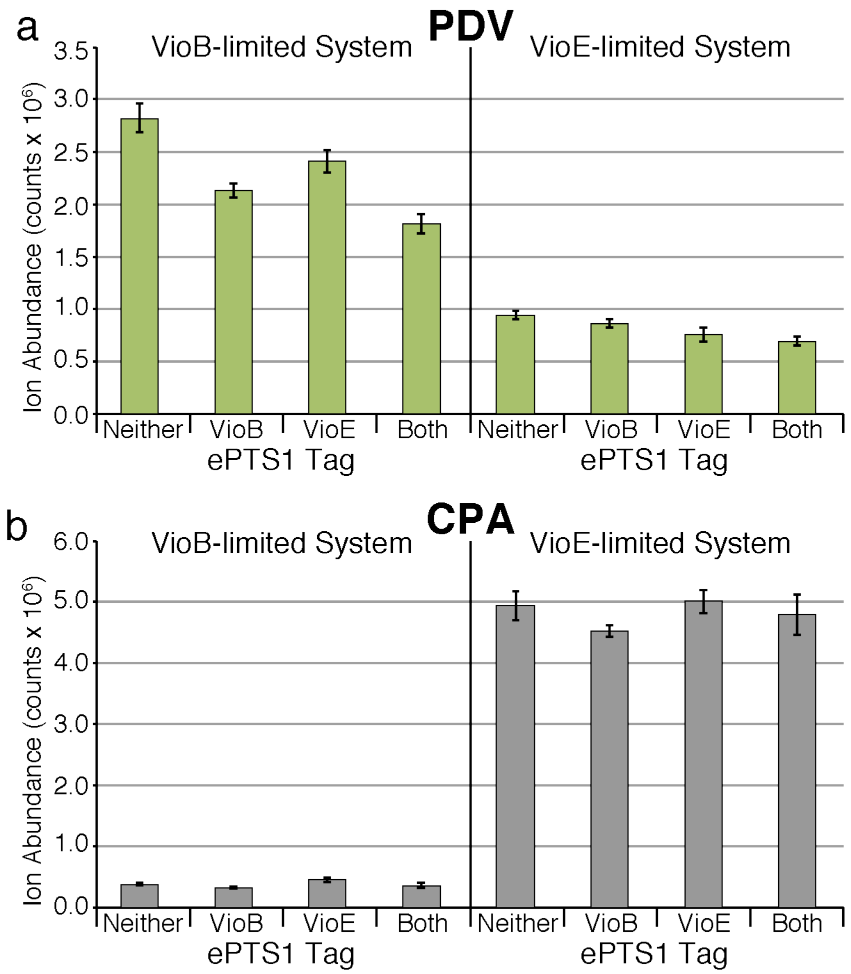

Supplementary Figure 15. *pex5Δ* import controls for Fig. 7. Measurement of CPA and PDV in yeast cytosolically expressing VioA while with or without ePTS1 tags on VioB and VioE in all combinations. All strains are *pex5Δ*, so no peroxisomal import is possible. Data is organized identically to Figure 7, with two different expression regimes shown: a regime where lower expression of VioB and higher expression of VioE cause VioB to be the limiting enzyme, and vice versa causing VioE to be the limiting enzyme. (**a**) Measurement of PDV extracted from cells. The ePTS1 tag seems to lead to decreased activity from the enzyme to which it is fused. (**b**) Measurement of CPA extracted from cells. Again, the ePTS1 tag seems to lead to a slight decrease in activity for each enzyme. Error bars are the mean ± s.d. of six biological replicates.

Linker Sequence	Net Charge	Relative PDV Production
LGRGRR	3	0.0955
SRARAG	2	0.0542
CRMWGK	2	0.0605
TRVKLV	2	0.0838
VRSTRG	2	0.0955
RVLNVK	2	0.0964
KDKSNK	2	0.1295
KLDRKV	2	0.1340
TYWIRF	1	0.0623
SVTVRS	1	0.0731
RSGAIC	1	0.0820
TGSYWR	1	0.0829
ALRLGI	1	0.0829
TTVATR	1	0.0901
GSRFTT	1	0.0928
RCVGIG	1	0.0946
SFALVR	1	0.0982
LATAKT	1	0.1000
VCCTCR	1	0.1035
WRREWG	1	0.1044
EKRVCM	1	0.1116
YGVKLT	1	0.1224
LTKVMT	1	0.1295
MRSNTA	1	0.1304
VRMVNT	1	0.1322
ACAKVS	1	0.1412
IYKAMC	1	0.1439
TTVSRV	1	0.1645
CVKVVT	1	0.1788
SLLIKS	1	0.1869
VAYKSS	1	0.2264
TRDKSC	1	0.2416
SKFCST	1	0.2506
KINLSA	1	0.2541
GGAKGG	1	0.2963
RTGVAG	1	0.3079
TSNKAF	1	0.3142
RYTGSF	1	0.3187
IFVGIF	0	0.0354
TSGLIY	0	0.0802
ASAVIA	0	0.0811
DSFRIT	0	0.0838
YVKWEV	0	0.0856
AASTVV	0	0.0892
SWFVVC	0	0.0919
GDSVRT	0	0.0982
DKFICM	0	0.1000
GLNSIT	0	0.1098
KGEWYW	0	0.1143
YVWSWA	0	0.1188
SDVSWR	0	0.1206
FVLLMM	0	0.1233
MVGSVS	0	0.1251
SMVIWV	0	0.1277
VYAWTF	0	0.1286
AALGCI	0	0.1322
SSILFY	0	0.1349
KDITVW	0	0.1358
FSAAVF	0	0.1367
YSYFGF	0	0.1385
AGVLTL	0	0.1394
VSSGIA	0	0.1421
CARYDM	0	0.1448
CYLTGW	0	0.1475
AYVTMA	0	0.1519
SATWWA	0	0.1528
YIMTTT	0	0.1528
LSLSMA	0	0.1555
ASSYLV	0	0.1564
WNYNCC	0	0.1591
YDSMRF	0	0.1618
CTSCTV	0	0.1627
LGFAGV	0	0.1699
MTAAVN	0	0.1788
FCSVSW	0	0.1815
FVSTLS	0	0.1824
SCYSLN	0	0.1860
CVKFET	0	0.1878
YITSAI	0	0.1887
TFLVTT	0	0.1950
YAGTVF	0	0.1968
AVISCA	0	0.2039
ENLKWS	0	0.2048
EACRSY	0	0.2057
VAMACA	0	0.2084
WGIYCC	0	0.2084
FSSCTV	0	0.2111
VSGTAA	0	0.2210
MGYINA	0	0.2264
TMMTAW	0	0.2308
TVSVWS	0	0.2335
LSTGCA	0	0.2434
ASTCVN	0	0.2479
IDTSSK	0	0.2524
GEKGIG	0	0.2541
AMCATS	0	0.2559
SSGGVS	0	0.2658
CGSIAY	0	0.2676
CVLSWG	0	0.2694
TTTAAV	0	0.2721
TVVYCG	0	0.2909
GGGVTC	0	0.2954
YAGSNT	0	0.2999
FKGVTE	0	0.3061
VVTTAT	0	0.3088
AAGGAW	0	0.3205
CVAVSF	0	0.3286
TTITAF	0	0.3375
WVWGAG	0	0.3393
ASRSSD	0	0.3420
AEKVEK	0	0.3438
ASFSNA	0	0.3501
SAAGAS	0	0.3501
GATAAT	0	0.3617
GCTAAT	0	0.3689
RTVAAD	0	0.3797
VTGCGT	0	0.3895
FAKTSD	0	0.4415
KFVSEA	0	0.4603
VGISGG	0	0.4603
GVCTGG	0	0.4639
ANNTSM	0	0.4666
VGGLGG	0	0.4693
SASSTS	0	0.5078
RSADAS	0	0.5473
AASGGG	0	0.5554
ASTSTT	0	0.5867
AGSGGG	0	0.6540
AVNSSG	0	0.6844
SSGGAG	0	0.7580
IIDFLV	-1	0.1358
TSEWLI	-1	0.1502
IVDCSG	-1	0.1959
YDSSVV	-1	0.2129
WEGYGC	-1	0.2201
WGYTIE	-1	0.2255
DALSCT	-1	0.2416
ASDSIA	-1	0.2541
FEIFAG	-1	0.2568
ELTGWT	-1	0.2568
GEGWMG	-1	0.2972
EMTTAA	-1	0.3017
WEVTAC	-1	0.3017
DTYVKE	-1	0.3079
NTEAVN	-1	0.3330
MVILES	-1	0.3393
MDWGSV	-1	0.3411
GEVSGI	-1	0.3716
SGFTTD	-1	0.3904
LTTIEV	-1	0.4119
AEWMYG	-1	0.4173
ENSYSF	-1	0.4290
FGAGAE	-1	0.4765
DAVSYS	-1	0.4836
WTAFSD	-1	0.5034
SSVYEN	-1	0.5087
MGAGYE	-1	0.5150
EYTTSS	-1	0.5213
VGGNAE	-1	0.5482
SCTDEK	-1	0.5536
ADISTN	-1	0.5616
FYNTEC	-1	0.5706
YTWSSD	-1	0.6127
SVNEGF	-1	0.6235
GSSADT	-1	0.6289
LSMTDF	-1	0.6584
ADVTSS	-1	0.6629
VGTDAS	-1	0.6683
SAYASD	-1	0.7024
EMICSG	-1	0.7176
GIVDGG	-1	0.7203
KNTEDT	-1	0.7571
NAAENS	-1	0.7660
NCAADG	-1	0.7750
SASTDG	-1	0.7822
SDGAGG	-1	0.7956
TNGDAS	-1	0.8153
LADSGG	-1	0.8270
TTCGTD	-1	0.8610
MAGLDG	-1	0.9068
ASNADG	-1	1.0816
GELGEV	-2	0.4801
GGDELC	-2	0.4989
GIVEYE	-2	0.5392
AGEIGE	-2	0.6387
TLDWDY	-2	0.6441
EECTGS	-2	0.6710
VVEESV	-2	0.6871
AGDLEY	-2	0.7409
TVDDMY	-2	0.7786
GGWVDE	-2	0.8091
TASEEF	-2	0.8252
NSDESI	-2	0.8404
TADAEA	-2	1.0332
YTSENE	-2	1.4527
AFCCDD	-2	1.7279
VDEECT	-3	0.7875
ESIEDA	-3	0.8010
ENENDC	-3	0.8306
EGAETE	-3	0.8404

Supplementary Table 1. PDV production linked to specific sequences from the 6-amino acid linker library. All 200 linker sequences measured in the library are shown here, along with a calculation of their net charge and the cells' PDV production relative to that of a defective peroxisomal import mutant (*pex5Δ*). PDV production was measured by bulk fluorescence at excitation/emission 535/585 nm. The data shown in this figure was used to generate Fig. 2A.

Strain #	Strain Name	Strain Parent	Plasmid Used	Description	Yeast Marker	Used in Figure
**1**	YJD051	BY4741	N/A	Openbiosystems *pex5Δ* :: KanMX (KO of YDR244W) Clone ID 3603 Catalog # YSC6273-201934299	Kan	
**2**	yWCD321	BY4741	pWCD1443	pTEF1-VioA,pTDH3-VioB	LEU2	
**3**	yWCD325	YJD051	pWCD1443	pTEF1-VioA,pTDH3-VioB	LEU2,Kan	
**4**	yWCD788	yWCD321	pWCD1134	URA3 Empty Vector	URA3,LEU2	1C
**5**	yWCD791	yWCD321	pWCD2424	pRPL18B-VioE-YFP-PTS1	URA3,LEU2	1C
**6**	yWCD797	yWCD325	pWCD2424	pRPL18B-VioE-YFP-PTS1	URA3,LEU2,Kan	1C
**7**	yZNR266	BY4741	pWCD2520	pRNR2-Pex11-CFP	LEU2	
**8**	yZNR267	YJD051	pWCD2520	pRNR2-Pex11-CFP	LEU2,Kan	
**9**	yWCD886	yZNR266	pWCD2424	pRPL18B-VioE-YFP-PTS1	URA3,LEU2	1D
**10**	yWCD887	yZNR267	pWCD2424	pRPL18B-VioE-YFP-PTS1	URA3,LEU2,Kan	1D
**11**	Library	yWCD321	pWCD2420L	pRPL18B-VioE-6xDNK-PTS1	URA3,LEU2	2A,S3,ST1
**12**	yWCD788	yWCD321	pWCD1134	URA3 Empty vector	URA3,LEU2	2B,S4,S5
**13**	yWCD789	yWCD321	pWCD2422	pREV1-VioE-YFP-PTS1	URA3,LEU2	2B,S5
**14**	yWCD790	yWCD321	pWCD2423	pRNR2-VioE-YFP-PTS1	URA3,LEU2	2B,S5
**15**	yWCD791	yWCD321	pWCD2424	pRPL18B-VioE-YFP-PTS1	URA3,LEU2	2B,S5
**16**	yWCD792	yWCD321	pWCD2425	pTEF1-VioE-YFP-PTS1	URA3,LEU2	2B,S5
**17**	yWCD793	yWCD321	pWCD2426	pTDH3-VioE-YFP-PTS1	URA3,LEU2	2B,S5
**18**	yWCD794	yWCD325	pWCD1134	URA3 Empty vector	URA3,LEU2,Kan	2B,S5
**19**	yWCD795	yWCD325	pWCD2422	pREV1-VioE-YFP-PTS1	URA3,LEU2,Kan	2B,S5
**20**	yWCD796	yWCD325	pWCD2423	pRNR2-VioE-YFP-PTS1	URA3,LEU2,Kan	2B,S5
**21**	yWCD797	yWCD325	pWCD2424	pRPL18B-VioE-YFP-PTS1	URA3,LEU2,Kan	2B,S4,S5
**22**	yWCD798	yWCD325	pWCD2425	pTEF1-VioE-YFP-PTS1	URA3,LEU2,Kan	2B,S5
**23**	yWCD799	yWCD325	pWCD2426	pTDH3-VioE-YFP-PTS1	URA3,LEU2,Kan	2B,S5
**24**	yWCD800	yWCD321	pWCD2427	pREV1-VioE-YFP-ePTS1	URA3,LEU2	2B,S5
**25**	yWCD801	yWCD321	pWCD2428	pRNR2-VioE-YFP-ePTS1	URA3,LEU2	2B,S5
**26**	yWCD802	yWCD321	pWCD2429	pRPL18B-VioE-YFP-ePTS1	URA3,LEU2	2B,S5
**27**	yWCD803	yWCD321	pWCD2430	pTEF1-VioE-YFP-ePTS1	URA3,LEU2	2B,S5
**28**	yWCD804	yWCD321	pWCD2431	pTDH3-VioE-YFP-ePTS1	URA3,LEU2	2B,S5
**29**	yWCD805	yWCD325	pWCD2427	pREV1-VioE-YFP-ePTS1	URA3,LEU2	2B,S5
**30**	yWCD806	yWCD325	pWCD2428	pRNR2-VioE-YFP-ePTS1	URA3,LEU2,Kan	2B,S5
**31**	yWCD807	yWCD325	pWCD2429	pRPL18B-VioE-YFP-ePTS1	URA3,LEU2,Kan	2B,S5
**32**	yWCD808	yWCD325	pWCD2430	pTEF1-VioE-YFP-ePTS1	URA3,LEU2,Kan	2B,S5
**33**	yWCD809	yWCD325	pWCD2431	pTDH3-VioE-YFP-ePTS1	URA3,LEU2,Kan	2B,S5
**34**	yZNR231	BY4741	pZNR0727	HIS3 Empty vector	HIS3	
**35**	yWCD543	BY4741	pWCD1873	pREV1-TEVprotease-CFP	HIS3	
**36**	yWCD544	BY4741	pWCD1874	pRNR2-TEVprotease-CFP	HIS3	
**37**	yWCD545	BY4741	pWCD1875	pRPL18B-TEVprotease-CFP	HIS3	
**38**	yWCD546	BY4741	pWCD1876	pTEF1-TEVprotease-CFP	HIS3	
**39**	yWCD547	BY4741	pWCD1877	pTDH3-TEVprotease-CFP	HIS3	
**40**	yWCD548	yZNR231	pJAG011	pRPL18B-RFP-TEVsite-YFP-PTS1	HIS3, URA3	3B,S6
**41**	yWCD549	yWCD543	pJAG011	pRPL18B-RFP-TEVsite-YFP-PTS1	HIS3, URA3	3B,S6
**42**	yWCD550	yWCD544	pJAG011	pRPL18B-RFP-TEVsite-YFP-PTS1	HIS3, URA3	S6
**43**	yWCD551	yWCD545	pJAG011	pRPL18B-RFP-TEVsite-YFP-PTS1	HIS3, URA3	3B,S6
**44**	yWCD552	yWCD546	pJAG011	pRPL18B-RFP-TEVsite-YFP-PTS1	HIS3, URA3	S6
**45**	yWCD553	yWCD547	pJAG011	pRPL18B-RFP-TEVsite-YFP-PTS1	HIS3, URA3	3B,S6
**46**	yWCD554	yZNR231	pWCD1879	pRPL18B-RFP-TEVsite-YFP-ePTS1	HIS3, URA3	3B,S6
**47**	yWCD555	yWCD543	pWCD1879	pRPL18B-RFP-TEVsite-YFP-ePTS1	HIS3, URA3	3B,S6
**48**	yWCD556	yWCD544	pWCD1879	pRPL18B-RFP-TEVsite-YFP-ePTS1	HIS3, URA3	S6
**49**	yWCD557	yWCD545	pWCD1879	pRPL18B-RFP-TEVsite-	HIS3, URA3	3B,S6

				YFP-ePTS1		
**50**	yWCD558	yWCD546	pWCD1879	pRPL18B-RFP-TEVsite-YFP-ePTS1	HIS3, URA3	S6
**51**	yWCD559	yWCD547	pWCD1879	pRPL18B-RFP-TEVsite-YFP-ePTS1	HIS3, URA3	3B,S6
**52**	yWCD560	yZNR231	pWCD1134	URA3 Empty vector	HIS3, URA3	S6
**53**	yWCD565	yWCD547	pWCD1134	URA3 Empty vector	HIS3, URA3	S6
**54**	yZNR142	BY4741	pZNR1271+R1	pPex5::pGal1	None	
**55**	yZNR232	yZNR142	pZNR0915	pRPL18B-RFP-ePTS1	None	S8
**56**	yZNR157	yZNR142	pZNR1261+R2	CDC14-ePTS1	None	4
**57**	yZNR158	yZNR142	pZNR1261+R3	CDC14-PTS1	None	4
**58**	yZNR147	yZNR142	pZNR1262+R4	CDC28-ePTS1	None	4
**59**	yZNR148	yZNR142	pZNR1262+R5	CDC28-PTS1	None	4
**60**	yZNR159	yZNR142	pZNR1263+R6	TYS1-ePTS1	None	4
**61**	yZNR160	yZNR142	pZNR1263+R7	TYS1-PTS1	None	4
**62**	yZNR153	yZNR142	pZNR1264+R8	SPC42-ePTS1	None	4
**63**	yZNR154	yZNR142	pZNR1264+R9	SPC42-PTS1	None	4
**64**	yZNR174	yZNR157	pZNR1326+R10	*pex14Δ*	None	4
**65**	yZNR175	yZNR158	pZNR1326+R10	*pex14Δ*	None	4
**66**	yZNR165	yZNR147	pZNR1326+R10	*pex14Δ*	None	4
**67**	yZNR166	yZNR148	pZNR1326+R10	*pex14Δ*	None	4
**68**	yZNR176	yZNR159	pZNR1326+R10	*pex14Δ*	None	4
**69**	yZNR177	yZNR160	pZNR1326+R10	*pex14Δ*	None	4
**70**	yZNR171	yZNR153	pZNR1326+R10	*pex14Δ*	None	4
**71**	yZNR172	yZNR154	pZNR1326+R10	*pex14Δ*	None	4
**72**	yWCD319	BY4741	pWCD1441	pTDH3-VioB pHHF2-VioE	LEU2	
**73**	yWCD320	BY4741	pWCD1442	pTEF1-VioA pHHF2-VioE	LEU2	
**74**	yWCD323	YJD051	pWCD1441	pTDH3-VioB pHHF2-VioE	LEU2, Kan	
**75**	yWCD324	YJD051	pWCD1442	pTEF1-VioA pHHF2-VioE	LEU2, Kan	
**76**	yWCD888	yWCD319	pWCD2526	pREV1-VioA-ePTS1	URA3, LEU2	6
**77**	yWCD889	yWCD319	pWCD2527	pRNR2-VioA-ePTS1	URA3, LEU2	6
**78**	yWCD890	yWCD319	pWCD2528	pRPL18B-VioA-ePTS1	URA3, LEU2	6
**79**	yWCD891	yWCD319	pWCD2529	pTEF1-VioA-ePTS1	URA3, LEU2	6
**80**	yWCD892	yWCD319	pWCD2530	pTDH3-VioA-ePTS1	URA3, LEU2	6
**81**	yWCD893	yWCD320	pWCD2531	pREV1-VioB-ePTS1	URA3, LEU2	6
**82**	yWCD894	yWCD320	pWCD2532	pRNR2-VioB-ePTS1	URA3, LEU2	6
**83**	yWCD895	yWCD320	pWCD2533	pRPL18B-VioB-ePTS1	URA3, LEU2	6
**84**	yWCD896	yWCD320	pWCD2534	pTEF1-VioB-ePTS1	URA3, LEU2	6
**85**	yWCD897	yWCD320	pWCD2535	pTDH3-VioB-ePTS1	URA3, LEU2	6
**86**	yWCD898	yWCD321	pWCD2536	pREV1-VioE-ePTS1	URA3, LEU2	6
**87**	yWCD899	yWCD321	pWCD2537	pRNR2-VioE-ePTS1	URA3, LEU2	6
**88**	yWCD900	yWCD321	pWCD2538	pRPL18B-VioE-ePTS1	URA3, LEU2	6
**89**	yWCD901	yWCD321	pWCD2539	pTEF1-VioE-ePTS1	URA3, LEU2	6
**90**	yWCD902	yWCD321	pWCD2540	pTDH3-VioE-ePTS1	URA3, LEU2	6
**91**	yWCD903	yWCD323	pWCD2526	pREV1-VioA-ePTS1	URA3, LEU2, Kan	6
**92**	yWCD904	yWCD323	pWCD2527	pRNR2-VioA-ePTS1	URA3, LEU2, Kan	6
**93**	yWCD905	yWCD323	pWCD2528	pRPL18B-VioA-ePTS1	URA3, LEU2, Kan	6
**94**	yWCD906	yWCD323	pWCD2529	pTEF1-VioA-ePTS1	URA3, LEU2, Kan	6
**95**	yWCD907	yWCD323	pWCD2530	pTDH3-VioA-ePTS1	URA3, LEU2, Kan	6
**96**	yWCD908	yWCD324	pWCD2531	pREV1-VioB-ePTS1	URA3, LEU2, Kan	6
**97**	yWCD909	yWCD324	pWCD2532	pRNR2-VioB-ePTS1	URA3, LEU2, Kan	6
**98**	yWCD910	yWCD324	pWCD2533	pRPL18B-VioB-ePTS1	URA3, LEU2, Kan	6
**99**	yWCD911	yWCD324	pWCD2534	pTEF1-VioB-ePTS1	URA3, LEU2, Kan	6
**100**	yWCD912	yWCD324	pWCD2535	pTDH3-VioB-ePTS1	URA3, LEU2, Kan	6
**101**	yWCD913	yWCD325	pWCD2536	pREV1-VioE-ePTS1	URA3, LEU2, Kan	6
**102**	yWCD914	yWCD325	pWCD2537	pRNR2-VioE-ePTS1	URA3, LEU2, Kan	6
**103**	yWCD915	yWCD325	pWCD2538	pRPL18B-VioE-ePTS1	URA3, LEU2, Kan	6
**104**	yWCD916	yWCD325	pWCD2539	pTEF1-VioE-ePTS1	URA3, LEU2, Kan	6
**105**	yWCD917	yWCD325	pWCD2540	pTDH3-VioE-ePTS1	URA3, LEU2, Kan	6
**106**	yWCD918	yWCD319	pWCD2541	pRPL18B-VioE-VioA-ePTS1	URA3, LEU2	S13B,S13C

**107**	yWCD919	yWCD321	pWCD2541	pRPL18B-VioE-VioA-ePTS1	URA3, LEU2	S13B,S13C
**108**	yWCD920	yWCD323	pWCD2541	pRPL18B-VioE-VioA-ePTS1	URA3, LEU2, Kan	S13B,S13C
**109**	yWCD921	yWCD325	pWCD2541	pRPL18B-VioE-VioA-ePTS1	URA3, LEU2, Kan	S13B,S13C
**110**	yZNR073	BY4741	pZNR0728	pCCW12-Nc_CDT1	LEU2	
**111**	yZNR075	BY4741	pZNR0521	LEU2 Empty vector	LEU2	
**112**	yZNR103	yZNR073	pZNR0873	pREV1-VioE-YFP-BGL-ePTS1	URA3, LEU2	5B
**113**	yZNR104	yZNR073	pZNR0872	pRNR2-VioE-YFP-BGL-ePTS1	URA3, LEU2	5B
**114**	yZNR105	yZNR073	pZNR0871	pRPL18B-VioE-YFP-BGL-ePTS1	URA3, LEU2	5B
**115**	yZNR106	yZNR073	pZNR0864	pTEF1-VioE-YFP-BGL-ePTS1	URA3, LEU2	5B
**116**	yZNR107	yZNR073	pZNR0870	pTDH3-VioE-YFP-BGL-ePTS1	URA3, LEU2	5B
**117**	yZNR108	yZNR073	pZNR0875	pTDH3-VioE-YFP-BGL-deadPTS1	URA3, LEU2	5B,S10
**118**	yZNR109	yZNR073	pZNR0868	pTEF1-VioE-YFP-BGL-deadPTS1	URA3, LEU2	5B
**119**	yZNR110	yZNR073	pZNR0876	pRPL18B-VioE-YFP-BGL-deadPTS1	URA3, LEU2	5B
**120**	yZNR111	yZNR073	pZNR0877	pRNR2-VioE-YFP-BGL-deadPTS1	URA3, LEU2	5B
**121**	yZNR112	yZNR073	pZNR0878	pREV1-VioE-YFP-BGL-deadPTS1	URA3, LEU2	5B
**122**	yZNR113	yZNR075	pZNR0875	pTDH3-VioE-YFP-BGL-deadPTS1	URA3, LEU2	S10
**123**	yZNR114	yZNR073	pWCD1134	URA3 Empty vector	URA3, LEU2	S10
**124**	yZNR115	yWCD321	pWCD1134	URA3 Empty vector	URA3, LEU2	S11
**125**	yZNR116	yWCD321	pZNR0873	pREV1-VioE-YFP-BGL-ePTS1	URA3, LEU2	S11
**126**	yZNR117	yWCD321	pZNR0872	pRNR2-VioE-YFP-BGL-ePTS1	URA3, LEU2	S11
**127**	yZNR118	yWCD321	pZNR0871	pRPL18B-VioE-YFP-BGL-ePTS1	URA3, LEU2	S11
**128**	yZNR119	yWCD321	pZNR0864	pTEF1-VioE-YFP-BGL-ePTS1	URA3, LEU2	S11
**129**	yZNR120	yWCD321	pZNR0870	pTDH3-VioE-YFP-BGL-ePTS1	URA3, LEU2	S11
**130**	yZNR121	yWCD321	pZNR0875	pTDH3-VioE-YFP-BGL-deadPTS1	URA3, LEU2	S11
**131**	yZNR122	yWCD321	pZNR0868	pTEF1-VioE-YFP-BGL-deadPTS1	URA3, LEU2	S11
**132**	yZNR123	yWCD321	pZNR0876	pRPL18B-VioE-YFP-BGL-deadPTS1	URA3, LEU2	S11
**133**	yZNR124	yWCD321	pZNR0877	pRNR2-VioE-YFP-BGL-deadPTS1	URA3, LEU2	S11
**134**	yZNR125	yWCD321	pZNR0878	pREV1-VioE-YFP-BGL-deadPTS1	URA3, LEU2	S11
**135**	yWCD591	BY4741	pWCD1932	pTDH3-VioA,pTDH3-VioB,pRPL18B-YFP-VioE-ePTS1	URA3	
**136**	yWCD592	YJD051	pWCD1932	pTDH3-VioA,pTDH3-VioB,pRPL18B-YFP-VioE-ePTS1	URA3, Kan	
**137**	yWCD593	BY4741	pWCD1933	pTDH3-VioA,pTDH3-VioB,pRPL18B-YFP-VioE-deadPTS1	URA3	
**138**	yWCD594	yWCD591	pWCD1934	HIS3 Empty vector	URA3, HIS3	S14
**139**	yWCD595	yWCD591	pWCD1935	pREV1-RFP-ePTS1	URA3, HIS3	S14
**140**	yWCD596	yWCD591	pWCD1936	pRNR2-RFP-ePTS1	URA3, HIS3	S14
**141**	yWCD597	yWCD591	pWCD1937	pRPL18B-RFP-ePTS1	URA3, HIS3	S14
**142**	yWCD598	yWCD591	pWCD1938	pTEF1-RFP-ePTS1	URA3, HIS3	S14
**143**	yWCD599	yWCD591	pWCD1924	pTDH3-RFP-ePTS1	URA3, HIS3	S14

**144**	yWCD600	yWCD591	pWCD1925	pTDH3-RFP-ePTS1,pCCW12-RFP_alt-ePTS1	URA3, HIS3	S14
**145**	yWCD601	yWCD591	pWCD1926	pTDH3-RFP-ePTS1,pCCW12-RFP_alt-ePTS1,pTDH3-RFP-ePTS1	URA3, HIS3	S14
**146**	yWCD602	yWCD592	pWCD1934	HIS3 Empty vector	URA3, HIS3, Kan	S14
**147**	yWCD603	yWCD592	pWCD1935	pREV1-RFP-ePTS1	URA3, HIS3, Kan	S14
**148**	yWCD604	yWCD592	pWCD1936	pRNR2-RFP-ePTS1	URA3, HIS3, Kan	S14
**149**	yWCD605	yWCD592	pWCD1937	pRPL18B-RFP-ePTS1	URA3, HIS3, Kan	S14
**150**	yWCD606	yWCD592	pWCD1938	pTEF1-RFP-ePTS1	URA3, HIS3, Kan	S14
**151**	yWCD607	yWCD592	pWCD1924	pTDH3-RFP-ePTS1	URA3, HIS3, Kan	S14
**152**	yWCD608	yWCD592	pWCD1925	pTDH3-RFP-ePTS1,pCCW12-RFP_alt-ePTS1	URA3, HIS3, Kan	S14
**153**	yWCD609	yWCD592	pWCD1926	pTDH3-RFP-ePTS1,pCCW12-RFP_alt-ePTS1,pTDH3-RFP-ePTS1	URA3, HIS3, Kan	S14
**154**	yWCD610	yWCD593	pWCD1934	HIS3 Empty vector	URA3, HIS3	S14
**155**	yWCD611	yWCD593	pWCD1935	pREV1-RFP-ePTS1	URA3, HIS3	S14
**156**	yWCD612	yWCD593	pWCD1936	pRNR2-RFP-ePTS1	URA3, HIS3	S14
**157**	yWCD613	yWCD593	pWCD1937	pRPL18B-RFP-ePTS1	URA3, HIS3	S14
**158**	yWCD614	yWCD593	pWCD1938	pTEF1-RFP-ePTS1	URA3, HIS3	S14
**159**	yWCD615	yWCD593	pWCD1924	pTDH3-RFP-ePTS1	URA3, HIS3	S14
**160**	yWCD616	yWCD593	pWCD1925	pTDH3-RFP-ePTS1,pCCW12-RFP_alt-ePTS1	URA3, HIS3	S14
**161**	yWCD617	yWCD593	pWCD1926	pTDH3-RFP-ePTS1,pCCW12-RFP_alt-ePTS1,pTDH3-RFP-ePTS1	URA3, HIS3	S14
**162**	yWCD516	BY4741	pWCD1824	pTDH3-VioA	LEU2	
**163**	yWCD517	YJD051	pWCD1824	pTDH3-VioA	LEU2, Kan	
**164**	yZNR233	yWCD516	pZNR1425	pRPL18B-VioB-YFP-ePTS1,pRNR2-VioE-RFP-ePTS1	URA3,LEU2	7
**165**	yZNR234	yWCD516	pZNR1427	pRPL18B-VioB-YFP-ePTS1,pRNR2-VioE-RFP	URA3,LEU2	7
**166**	yZNR235	yWCD516	pZNR1429	pRPL18B-VioB-YFP,pRNR2-VioE-RFP-ePTS1	URA3,LEU2	7
**167**	yZNR236	yWCD516	pZNR1431	pRPL18B-VioB-YFP,pRNR2-VioE-RFP	URA3,LEU2	7
**168**	yZNR237	yWCD517	pZNR1425	pRPL18B-VioB-YFP-ePTS1,pRNR2-VioE-RFP-ePTS1	URA3,LEU2,Kan	S15
**169**	yZNR238	yWCD517	pZNR1427	pRPL18B-VioB-YFP-ePTS1,pRNR2-VioE-RFP	URA3,LEU2,Kan	S15
**170**	yZNR239	yWCD517	pZNR1429	pRPL18B-VioB-YFP,pRNR2-VioE-RFP-ePTS1	URA3,LEU2,Kan	S15
**171**	yZNR240	yWCD517	pZNR1431	pRPL18B-VioB-YFP,pRNR2-VioE-RFP	URA3,LEU2,Kan	S15
**172**	yZNR241	yWCD516	pZNR1433	pTEF1-VioB-YFP-ePTS1,pREV1-VioE-RFP-ePTS1	URA3,LEU2	7
**173**	yZNR242	yWCD516	pZNR1435	pTEF1-VioB-YFP-ePTS1,pREV1-VioE-RFP	URA3,LEU2	7
**174**	yZNR243	yWCD516	pZNR1437	pTEF1-VioB-YFP,pREV1-VioE-RFP-ePTS1	URA3,LEU2	7
**175**	yZNR244	yWCD516	pZNR1439	pTEF1-VioB-YFP,pREV1-VioE-RFP	URA3,LEU2	7
**176**	yZNR245	yWCD517	pZNR1433	pTEF1-VioB-YFP-ePTS1,pREV1-VioE-RFP-ePTS1	URA3,LEU2,Kan	S15
**177**	yZNR246	yWCD517	pZNR1435	pTEF1-VioB-YFP-ePTS1,pREV1-VioE-RFP	URA3,LEU2,Kan	S15
**178**	yZNR247	yWCD517	pZNR1437	pTEF1-VioB-YFP,pREV1-	URA3,LEU2,Kan	S15

				VioE-RFP-ePTS1		
**179**	yZNR248	yWCD517	pZNR1439	pTEF1-VioB-YFP,pREV1-VioE-RFP	URA3,LEU2,Kan	S15

Supplementary Table 2. List of yeast strains used in this work. Strains were made by chromosomal integration of plasmids directly into the parent strain, except when a repair partner is noted as in Strain 54 and Strains 56-71. These strains were cotransformed with the plasmid and repair DNA listed, plated, and cured of the Cas9 plasmid once CRISPR genomic modification was confirmed. Also note that all references to the red fluorescent protein mKate2 are abbreviated as RFP, Venus is referred to as YFP, and mTurquoise2 is listed as CFP. “S” indicates a supplementary figure; “ST” indicates a supplementary table. All sequences of plasmids and repair DNA listed are provided as Genbank format files in the zip file Supplementary Data 1.

Supplementary References

1. Antonenkov, V. D., Mindthoff, S., Grunau, S., Erdmann, R. & Hiltunen, J. K. An involvement of yeast peroxisomal channels in transmembrane transfer of glyoxylate cycle intermediates. *Int. J. Biochem. Cell Biol.* **41,** 2546–2554 (2009).
2. Grunau, S. *et al.* Channel-forming activities of peroxisomal membrane proteins from the yeast Saccharomyces cerevisiae. *FEBS J.* **276,** 1698–1708 (2009).
3. Meinecke, M. *et al.* The peroxisomal importomer constitutes a large and highly dynamic pore. *Nat. Cell Biol.* **12,** 273–277 (2010).
4. Antonenkov, V. D., Sormunen, R. T. & Hiltunen, J. K. The rat liver peroxisomal membrane forms a permeability barrier for cofactors but not for small metabolites in vitro. *J. Cell Sci.* **117,** 5633–5642 (2004).
5. Kuga, S. Pore size distribution analysis of gel substances by size exclusion chromatography. *J. Chromatogr. A* **206,** 449–461 (1981).
6. Kurath, S. F. & Bump, D. D. Hydrodynamic friction coefficients for cellodextrins in water. *J. Polym. Sci. A* **3,** 1515–1526 (1965).
7. Ihnat, M. & Goring, D. A. I. Shape of the cellodextrins in aqueous solution at 25 °C. *Can. J. Chem.* **45,** 2353–2361 (1967).
